# Efficient and Stable Inverted Perovskite Solar Modules Enabled by Solid–Liquid Two-Step Film Formation

**DOI:** 10.1007/s40820-024-01408-2

**Published:** 2024-05-02

**Authors:** Juan Zhang, Xiaofei Ji, Xiaoting Wang, Liujiang Zhang, Leyu Bi, Zhenhuang Su, Xingyu Gao, Wenjun Zhang, Lei Shi, Guoqing Guan, Abuliti Abudula, Xiaogang Hao, Liyou Yang, Qiang Fu, Alex K.-Y. Jen, Linfeng Lu

**Affiliations:** 1 Graduate School of Science and Technology, Hirosaki University, 3-Bunkyocho, Hirosaki, 036-8561 Japan; 2 Department of Materials Science and Engineering, Department of Chemistry, Hong Kong Institute for Clean Energy, City University of Hong Kong Kowloon, Hong Kong, 999077 People’s Republic of China; 3 The Interdisciplinary Research Center, Shanghai Advanced Research Institute, Chinese Academy of Sciences, Shanghai, 201210 People’s Republic of China; 4 Shanghai Synchrotron Radiation Facility (SSRF), Zhangjiang Lab, Shanghai Advanced Research Institute, Chinese Academy of Sciences, Shanghai, 201204, People’s Republic of China; 5 JINNENG Clean Energy Technology Ltd., Jinzhong, 030300 Shanxi People’s Republic of China; 6 Hangzhou Zhongneng Photoelectricity Technology Co., Ltd., Hangzhou, 310018 People’s Republic of China; 7 College of Chemical Engineering and Technology, Taiyuan University of Technology, Taiyuan, 030024 People’s Republic of China; 8 Institute of Regional Innovation, Hirosaki University, 3-Bunkyocho, Hirosaki, 036-8561 Japan

**Keywords:** Inverted perovskite solar cells, Perovskite solar modules, Two-step film formation, Crystallization, Defect passivation

## Abstract

A considerable efficiency gap exists between large-area perovskite solar modules and small-area perovskite solar cells. The control of forming uniform and large-area film and perovskite crystallization is still the main obstacle restricting the efficiency of PSMs. In this work, we adopted a solid–liquid two-step film formation technique, which involved the evaporation of a lead iodide film and blade coating of an organic ammonium halide solution to prepare perovskite films. This method possesses the advantages of integrating vapor deposition and solution methods, which could apply to substrates with different roughness and avoid using toxic solvents to achieve a more uniform, large-area perovskite film. Furthermore, modification of the NiO_x_/perovskite buried interface and introduction of Urea additives were utilized to reduce interface recombination and regulate perovskite crystallization. As a result, a large-area perovskite film possessing larger grains, fewer pinholes, and reduced defects could be achieved. The inverted PSM with an active area of 61.56 cm^2^ (10 × 10 cm^2^ substrate) achieved a champion power conversion efficiency of 20.56% and significantly improved stability. This method suggests an innovative approach to resolving the uniformity issue associated with large-area film fabrication.

**Supplementary Information:**

The online version contains supplementary material available at 10.1007/s40820-024-01408-2.

## Highlights

- High-quality large-area perovskite films are prepared using a solid–liquid two-step film formation method combined with CsBr modification for the buried interface and Urea additive for perovskite crystallization.
- The inverted perovskite solar modules’ performance is enhanced to 20.56% in 61.56 cm^2^ with improved stability.

**Supplementary Information:**

The online version contains supplementary material available at 10.1007/s40820-024-01408-2.

## Graphical abstract

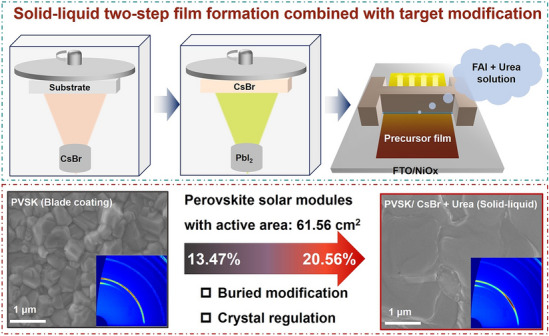

## Introduction

Perovskite solar cells (PSCs) have recently achieved power conversion efficiency (PCE) of 26.1% for single-junction cells and 33.9% for crystalline silicon tandem solar cells (TSCs) [1, 2]. While perovskite photovoltaics have entered the preliminary stages of commercialization, there are two obstacles to their commercialization including performance loss due to area amplification and challenges in fabricating high-quality, large-area, and stable perovskite films [3–5]. Small-area devices with the highest efficiency are often prepared in the laboratory through spin coating [6]. However, this is inadaptable for preparing large-area PSCs [7]. Currently, the fabrication of large-area perovskite films is divided into solution and vapor processes [8]. Solution-based preparation methods include blade coating, slot-die coating, spray coating, inkjet printing, and screen printing. These methods have the advantages of cost-effectiveness, quick preparation, and good compatibility for roll-to-roll production [9–12]. However, these processes often introduce toxic solvents, and it is difficult to control the processing parameters [13]. In addition, it is challenging to prepare a uniform film layer on textured substrates and uneven substrates [14]. These shortcomings limit the development of the solution-based method.

Vapor-based methods, including thermal evaporation (TE) and chemical vapor deposition, produce perovskite films under vacuum conditions without solvents [15]. The vapor deposition has the advantages of forming controlled thickness, morphology, and uniformity of perovskite films and can be conformally deposited on substrates with different roughness [16]. However, there is a substantial difference in the vaporization temperatures between lead iodide (PbI_2_) and amine salts. It is challenging to control PbI_2_ and amine salts to be evaporated at a reasonable deposition rate and a precise ratio [17]. Additionally, vapor deposition has drawbacks, such as low utilization of precursor materials, slower production rate, and higher energy consumption than the solution process [16]. During the TE process, FAI and MAI may decompose to produce corrosive gases such as HI, causing extensive damage to the equipment [18]. Therefore, it would be ideal if techniques that combine solution and vapor deposition methods could be developed to fabricate high-quality large-area perovskite films [19].

In this work, a solid–liquid two-step film formation method was adopted to deposit PbI_2_ through a vapor process. Then, the organic ammonium halide solution was blade-coated onto the PbI_2_ film to form the perovskite film. Compared with the traditional solution method, this method can accommodate substrates with different roughness, avoid toxic solvents, and achieve a more uniform large-area perovskite film. Furthermore, the CsBr modification can be used to reduce interface recombination for the NiO_x_/perovskite buried interface. The introduction of Urea in organic ammonium halide solution can regulate the perovskite crystallization and induce secondary growth of perovskite, obtaining a large-area perovskite film with large grains, less pinholes, and fewer defects. Based on the optimized perovskite film, we achieved a PCE of 20.56% in a perovskite solar module with an active area of 61.56 cm^2^ (10 × 10 cm^2^ substrate). Most importantly, the resulting module shows substantially improved environmental, light, and thermal stability.

## Experimental Section

### Materials

All the chemicals were purchased from commercial businesses without further purification: Formamidinium iodide (FAI) (98%, Greatcell Solar Materials Pty Ltd), Lead iodide (PbI_2_) (99.99%, Zhejiang Yitai Technology Co., Ltd.), Urea (99.999%, Aladdin), Bathocuproine (BCP) (> 99.0%, TCI), Fullerene (C_60_) (99.9%, Tanfeng Tech. Inc.), and Copper particle (Cu) (99.999%, Fuzhou Yingfeixun Photoelectric Technology Co., Ltd). Isopropanol (IPA) was purchased from Sigma-Aldrich. Cesium bromide (CsBr) (99.9%) and methylammonium chloride (MACl) (99.5%) were purchased from Xi’an Polymer Light Technology in China. All the materials are stored in the nitrogen-filled glove box to avoid the water.

### Perovskite Photovoltaic Mini-Modules Fabrication

For the large-size perovskite modules, laser etching, including P1, P2, and P3 processes, was conducted by a nanosecond laser (ZNLB-22V1-LW300). Before use, the fluorine-doped tin oxide glass (FTO) was cleaned with ultraviolet ozone for 15 min. The following procedures were fabricated on the pre-patterned large FTO glass substrates. The mini-modules were fabricated on the pre-patterned large FTO glass substrates (10 × 10 cm^2^). For the P1 process, 10 × 10 cm^2^ size FTO substrates were patterned with a scribing width of 35 μm with 11-strip connected in series. The NiO_x_ films were prepared by magnetron sputtering at 9 × 10^–4^ Pa. The power was controlled at 500 W for 300 s, and the thickness was about 25 nm. CsBr (15 nm) and PbI_2_ (300 nm) were deposited sequentially by thermal evaporation on the NiO_x_ substrates. Subsequently, the solution of FAI: MACl (110 mg:11 mg) in the absence or the presence of Urea in 1 mL IPA was blade-coated onto the above PbI_2_-covered FTO glass substrates at a movement speed of 15 mm s^−1^ in air. The N_2_ knife worked at 0.5 kaf cm^−2^ during blade coating. Then, the film was annealed at 150 °C for 20 min in air with a relative humidity of 40 ± 5%. Afterward, 25-nm C_60_, 5-nm BCP, and 240-nm copper were sequentially deposited sequentially using thermal evaporation under a high vacuum (≤ 8 × 10^−4^ Pa). For the P3 process, the Cu layer was scribed with a 95 µm width. A full structure of the large-size PSC is shown in Fig. S13. The fabricated modules typically have 11 sub-cells, each with a width of 7.33 mm. The total dead width was 0.332 mm, giving a GFF of 95.47%.

The mini-modules were fabricated on the pre-patterned large FTO glass substrates (10 × 10 cm^2^, P1 width 35 μm) following the same procedure as the solar cells. The fabricated modules typically have 11 sub-cells, each with a width of 7.33 mm. The laser scribing was performed twice with a laser marker. The final widths of P2 and P3 were measured to be 116 and 95 μm, respectively. The total scribing line width was 0.332 mm, giving a GFF of 95.47%.

### Solar Cell Characterization

The current density–voltage (*J–V*) characteristics of the mini-modules were measured using a Keithley 2400 Source Meter under standard AM1.5 G illumination, and the light intensity was calibrated using a standard silicon reference cell (Newport, Oriel Sol3Atm). The *J-V* curves were measured by forward scan (− 0.1 to 13 V) and reverse scan (13 to − 0.1 V). External quantum efficiency (EQE) spectra were obtained with a PVE300-IVT QE measurement kit by focusing a monochromatic light beam onto the devices.

## Results and Discussion

### Design Principle and Analysis

**Fig. 1:**
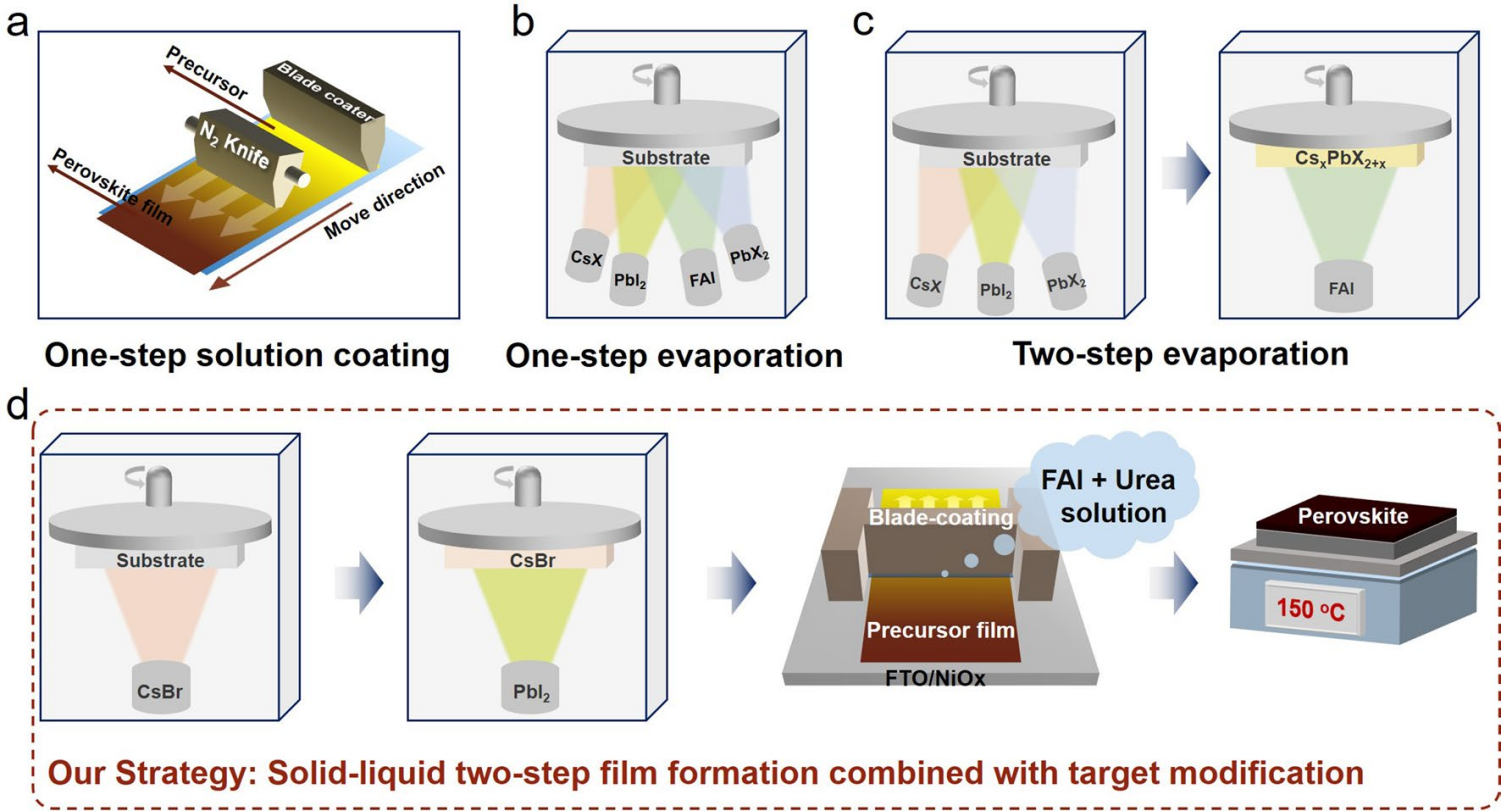
Illustrations of common large-area perovskite deposition methods, including **a** solution-processing techniques (blade coating, slot die coating, and spray coating), **b** one-step thermal evaporation, and **c** two-step thermal evaporation techniques. **d** The strategy of the perovskite film preparation in this work: solid–liquid two-step film formation combined with target modification

Blade coating, slot-die coating, and spray coating have all been proven to be very effective for solution processing of large-sized perovskite films (Fig. 1a). Nonetheless, these methods often require the use of toxic solvents, such as N,N-dimethylformamide (DMF) [20]. Recently, organic–inorganic halide perovskites have been successfully deposited through either one-step thermal evaporation (TE) or multi-step TE. A schematic illustration of various TE methods is shown in Fig. 1b, c. TE has shown several advantages, such as precisely controlled growth rate of various perovskite precursors with highly reproducible film thickness, large-area film uniformity, minimal material waste, and good adaptability to different substrates, such as flexible plastic substrates and textured silicon. However, the residual HI produced through decomposed ammonium salts will cause severe equipment damage, leading to equipment failure. In addition, FAI must diffuse into PbI_2_ first before a chemical reaction can occur to form a perovskite film [21]. Therefore, the unreacted PbI_2_ often strongly impacts the photoelectric performance of perovskite films. In contrast, the employment of a solid–liquid two-step film-forming process (Fig. 1d) can better prepare uniformly thick perovskite films on uneven substrate surfaces than the solution process. It can solve the problems of slow growth rate, low material utilization rate, and poorer material performance in vacuum evaporation. Moreover, it can solve the problem of forming residual PbI_2_ in perovskite materials due to incomplete reactions. Therefore, it is not only suitable for preparing large-area PSMs but also for preparing perovskite/crystalline silicon tandem solar cells on textured silicon.

### Regulation of Buried Interface and Lead Iodide Layer

**Fig. 2:**
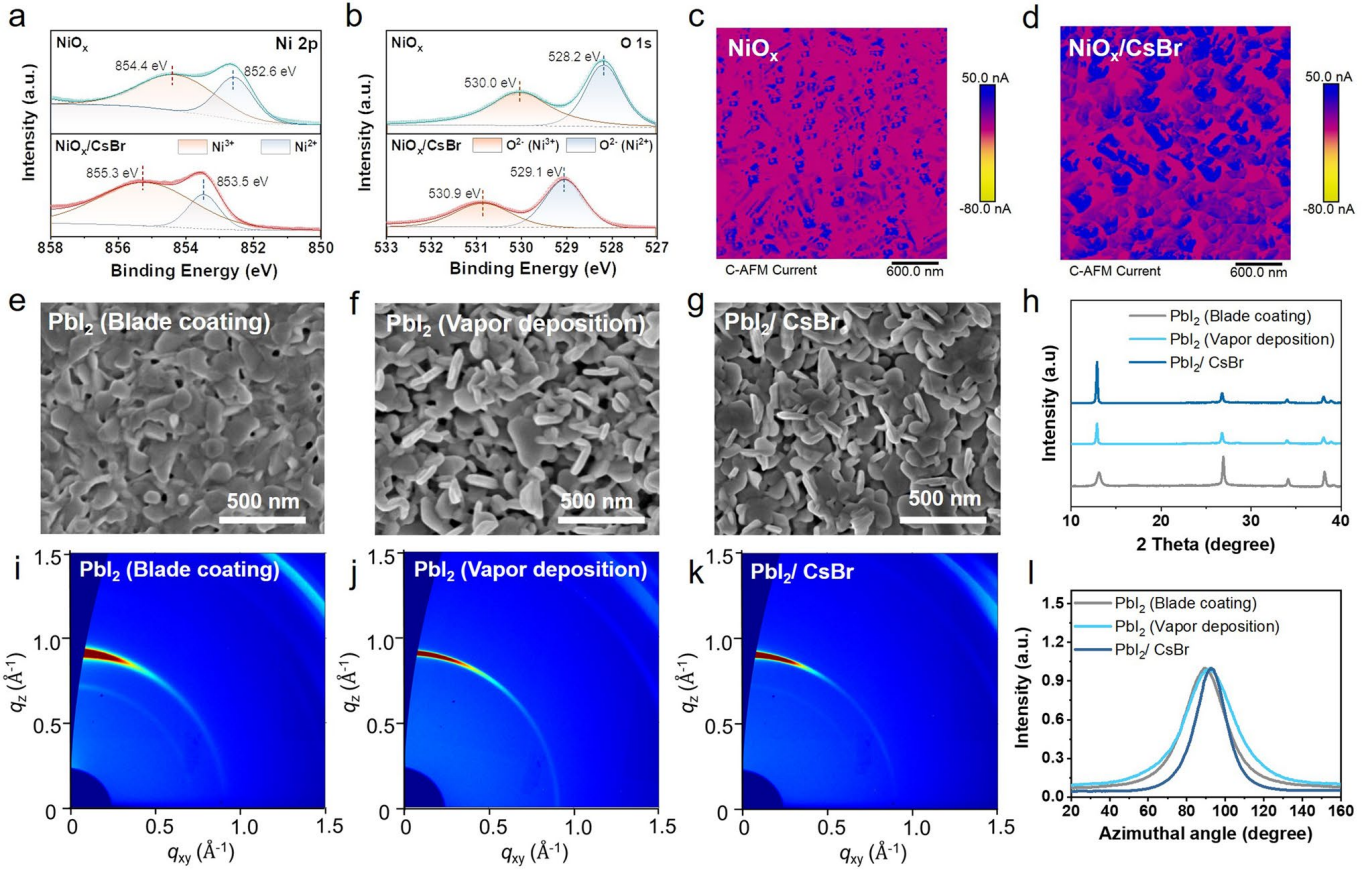
XPS spectra of **a** Ni 2*p* and **b** O 1*s* regions of NiO_x_ and NiO_x_/CsBr film. **c, d** c-AFM images of **c** NiO_x_ and **d** NiO_x_/CsBr film. The SEM images of PbI_2_ prepared by **e** blade coating and **f** vapor deposition, and **g** PbI_2_/CsBr film. **h** XRD patterns of PbI_2_ films (deposited by blade coating and vapor) and PbI_2_/CsBr film. **i-k** 2D GIWAXS patterns of corresponding PbI_2_ films. **l** Normalized integrated intensities of the PbI_2_ (001) planes are plotted as a function of the azimuthal angle

Nickel oxide (NiO_x_) is widely adopted as a hole transport material due to its low cost, stability, and availability [22]. However, given the high reactivity of organic cations, NiO_x_ may cause the decomposition of perovskites due to the presence of under-coordinated metal cation sites (like Ni^≥3+^) and dangling bonds at NiO_x_ surfaces [23]. To date, abundant assorted organic interlayers, such as Lewis acid–base organic salts [24], dye molecules [25], and self-assembly monolayers [26, 27], have been devoted to suppressing the defects at NiO_x_/perovskite interface. To simultaneously modify the NiO_x_ surface and induce the crystal growth of perovskite, we introduced the inorganic CsBr layer before the deposition of PbI_2_ (Fig. 1d). Through atomic force microscopy (AFM) and scanning electron microscopy (SEM) images, the CsBr exhibited “isolated islands” morphology on the NiO_x_ (Fig. S1). X-ray photoelectron spectroscopy (XPS) was further applied to investigate the electronic state on the NiO_x_ surface. The Ni 2*p* spectra of the pristine NiO_x_ and NiO_x_/CsBr films are shown in Fig. 2a, which can be de-convoluted into Ni^2+^ and Ni^3+^. The Ni^2+^ was characteristic of the standard Ni–O octahedral bonding configuration of the cubic NiO_x_ rock salt and the Ni^3+^ corresponded to Ni_2_O_3_ or NiOOH [28]. As shown in Fig. 2b, the binding energy of Ni^2+^ and Ni^3+^ shifted to high-binding energy after being modified with CsBr (852.6 to 853.5 eV for Ni^2+^ and 854.4 to 855.3 eV for Ni^3+^). The peaks of O 1*s*, which were banded with different Ni species, showed the same trend. These core-level peak shifts suggested a strong interaction between NiO_x_ and CsBr due to ion doping and surface passivation [26]. NiO_x_/CsBr exhibited a higher Ni^3+^/Ni^2+^ ratio (3.89) than pristine NiO_x_ (2.12), indicating the enhanced concentration of Ni^3+^, which contribute to the improvement of the conductivity and *p*-type property of NiO_x_, The results are verified by the enhanced current evaluated by conductive AFM (c-AFM) in Fig. 2c, d [29, 30].

Well-controlled crystallization of PbI_2_ precursor film is vital to enhance the quality of the final perovskite film [18, 31]. As shown in Fig. 2e, the blade-coated PbI_2_ showed a relatively rough morphology. In contrast, the vapor-deposited PbI_2_ films with different deposition rates exhibited a two-dimensional sheet-like structure with dense and uniformly distributed pores, which facilitated the subsequent diffusion of organic halides and solid-state crystallization reaction with PbI_2_ (Figs. 2f and S2). In addition, the PbI_2_ and PbI_2_/CsBr films prepared by vapor deposition showed better crystallinity with enlarged grain size than PbI_2_ of blade coating (Fig. 2f, g), compliance with the enhanced intensity of the (001) peaks in the X-ray diffraction (XRD) measurements (Fig. 2h). The grazing-incidence wide-angle X-ray scattering (GIWAXS) was performed to investigate the variation in the crystallographic orientations of the different PbI_2_ films. The 2D GIWAXS patterns and corresponding line-cut profiles are shown in Figs. 2i-k and S3, which accorded with the XRD results mentioned above. Furthermore, the azimuth angles were extracted from the 2D patterns of the (001) diffraction at the scattering vector q≈0.9 Å^−1^ (Fig. 2l). The azimuth angle of the blade-coated PbI_2_ and vapor-deposited PbI_2_ showed the broad distribution ranging from 40° to 140° with full-width at half-maximum (FWHM) of 26.4° and 31.4°, respectively, indicating their relatively random crystallographic orientation [32]. However, a sharper peak with the FWHM of 23.3° verified the preferred out-of-plane orientation along the (001) facet for the PbI_2_/CsBr film [18, 33]. Overall, the vapor-deposited PbI_2_ can easily form a two-dimensional sheet morphology with increased crystallinity, which was beneficial to the penetration of FAI. The buried modification of CsBr not only passivated NiO_x_ surface defects to reduce recombination but also regulated the crystal growth orientation of the upper PbI_2_.

### Morphology Modulation of Urea Additive

**Fig. 3:**
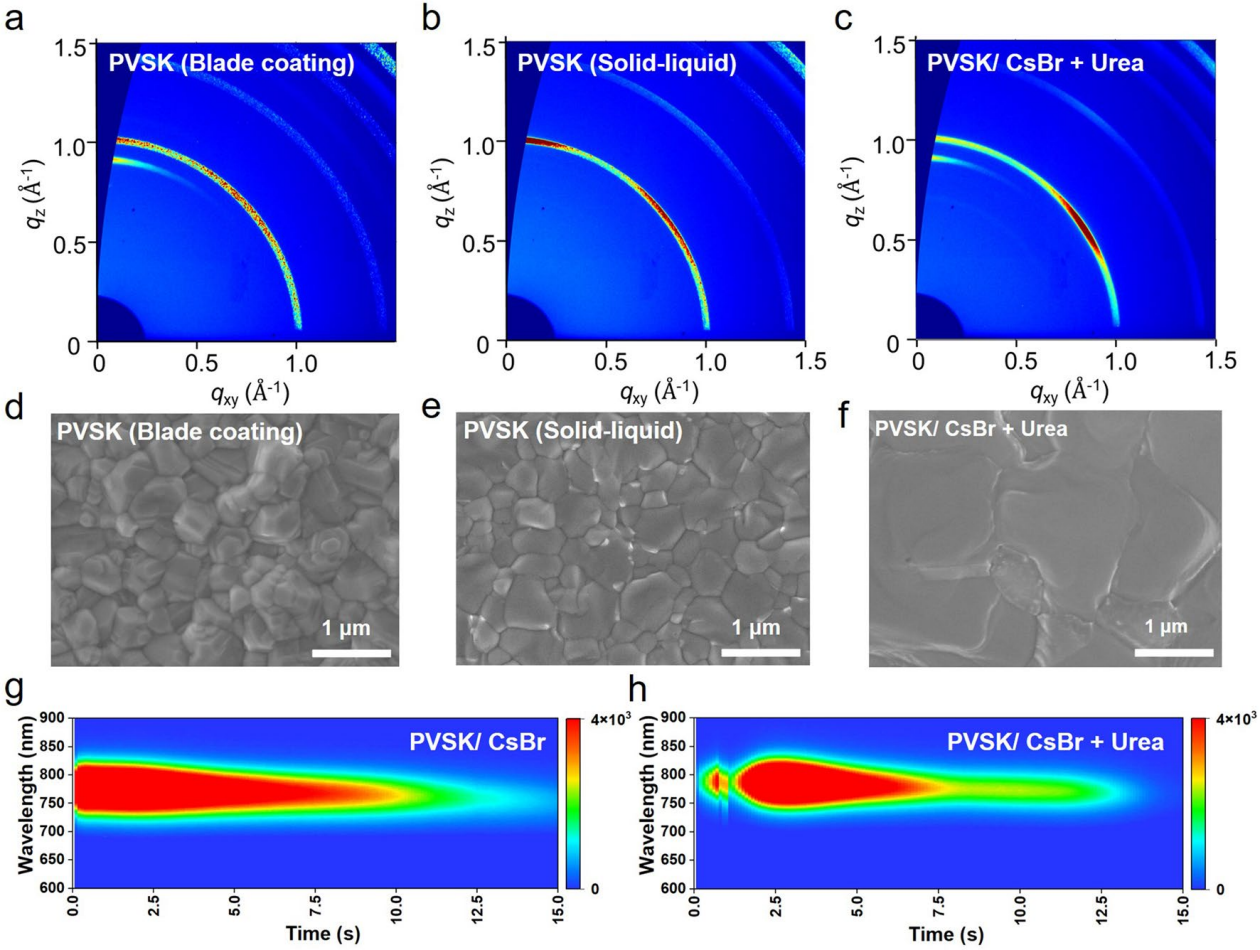
**a, b** 2D GIWAXS patterns of pristine perovskite film (prepared by blade coating and solid–liquid deposition), and **c** the perovskite film modified with CsBr + Urea. Note that the modified perovskite films were prepared using a solid–liquid strategy. **d-f** SEM images of corresponding pristine and modified perovskite films. Heat maps of in situ PL for **g** PVSK/CsBr and **h** PVSK/CsBr + Urea

To obtain high-quality large-area perovskite film, we introduced functional additive (Urea) with Lewis base groups into the FAI solution to regulate the crystal growth of the perovskite film. Furthermore, we performed the GIWAXS and SEM measurements to evaluate the effects of different deposition methods, buried interface modification and additive engineering on the perovskite growth, including grain size and crystal orientation. As shown in Fig. 3a-c, the solid–liquid deposited perovskite films exhibited higher diffraction intensity compared with blade-coated perovskite film according to the integrated XRD patterns from Fig. S4, indicating the better crystallinity of the vapor-deposited film. The intensities of the diffraction ring at the scattering vector *q*≈1.0 Å^−1^ along the azimuthal direction for each of the films and the normalized curves are plotted in Fig. S5. The orientation distribution of the perovskite film based on blade coating and vapor deposition showed similar three-peak patterns around 40°, 90°, and 140°, indicating two preferential orientations with the (111) and (100) planes parallel to the substrate [33]. By modified by CsBr + Urea, the intensities of the peaks around an azimuthal angle of 90° based on PVSK/CsBr + Urea significantly decrease, suggesting that the synergistic effect of CsBr and Urea can effectively suppress the crystal growth in the mode with the (100) planes [34]. Moreover, the SEM images revealed that the morphology of perovskite films prepared by vapor deposition (Fig. 3e, f) was smoother with larger grain size than that of the blade-coating film (Fig. 3d), especially for the perovskite film with CsBr + Urea, which can destabilize smaller grains and facilitate the growth of larger grains [35].

**Fig. 4:**
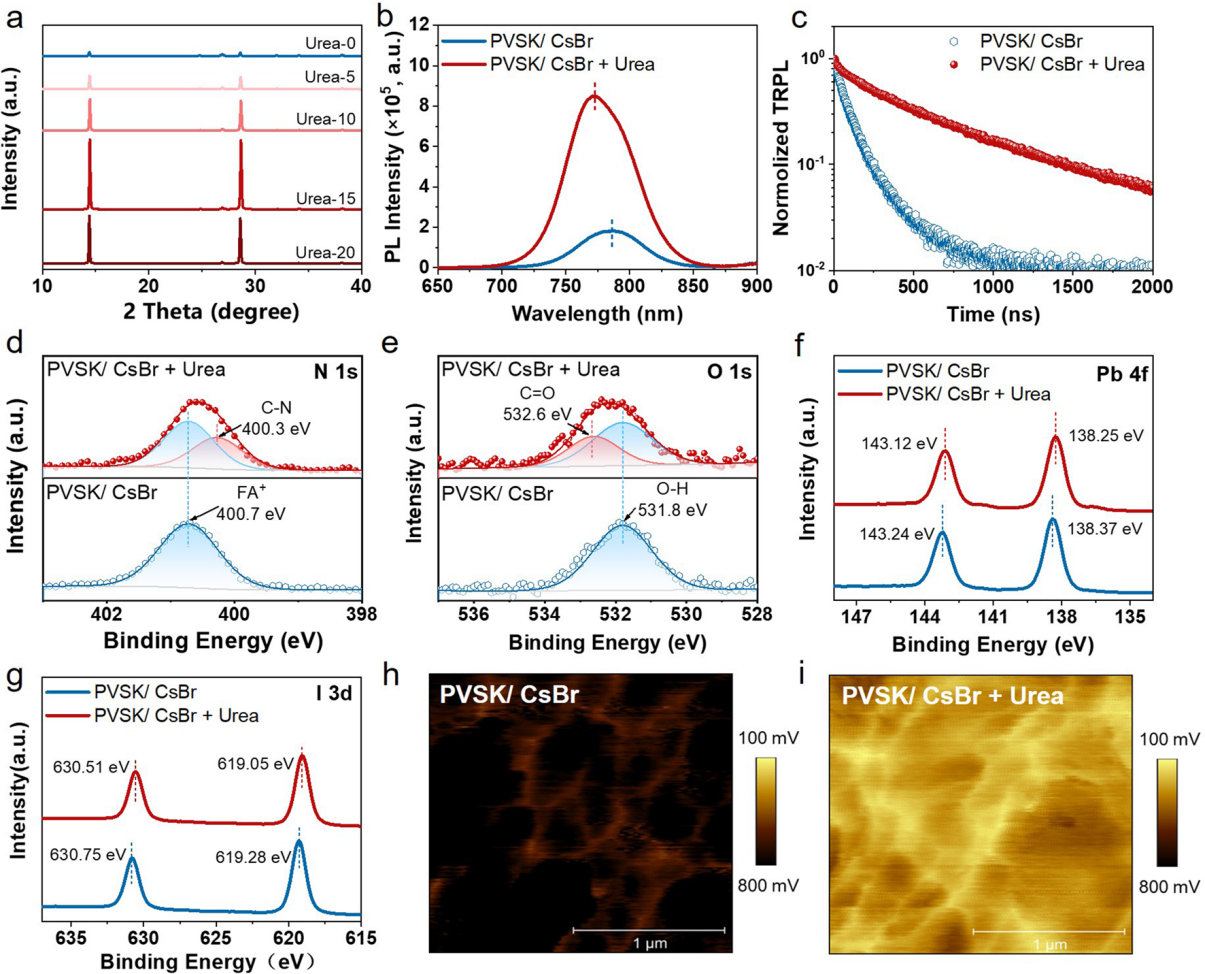
**a** XRD patterns of perovskite films modified with buried CsBr and vary concentrate Urea. **b** PL and **c** TRPL spectra of the PVSK/CsBr and PVSK/CsBr + Urea films. XPS spectra of **d** N 1*s*, **e** O 1*s*, **f** Pb 4*f*, and **g** I 3*d* for PVSK/CsBr and PVSK/CsBr + Urea films. The KPFM images of **h** PVSK/CsBr and **i** PVSK/CsBr + Urea films

The SEM images and grain size distribution of PVSK/CsBr + Urea films with varied concentrations are shown in Fig. S6. The optimal additive concentration of Urea was 15 mg mL^−1^, with an average grain size larger than 2 μm and the highest XRD intensity (Fig. 4a). These results suggested that the introduced Urea can promote large grain growth, contributing to decreased grain boundaries and reduced bulk defects of perovskite film. To further study the function of Urea in the perovskite crystallization process, *in situ* photoluminescence (PL) measurements were also taken (Fig. 3g, h). After blade coating the FAI solution onto a PbI_2_ substrate, the sample was immediately transferred to a hot plate at 150 °C and monitored through in situ PL. The heat maps of the PL spectra during the annealing process before and after adding Urea into the FAI solution are presented in Fig. 3g, h. We observed that the PVSK/CsBr + Urea sample achieved the maximum PL intensity within the first 2.6 s of annealing. In comparison, the PVSK/CsBr sample reached maximum PL intensity at 0.3 s (Fig. S7). This phenomenon suggested that Urea can form an intermediate nucleus, leading to slower crystallization [36]. In addition, the PL intensity of the maximum peak had a process of enhancement within 2.5 s for the PVSK/CsBr + Urea sample, indicating that Urea may cause secondary crystal growth of the perovskite film.

### Interaction Between Perovskite and Additive

Furthermore, the steady-state and time-resolved PL measurements were employed to understand the effect of the buried CsBr layer and Urea additive on the carrier recombination dynamics. As shown in Figs. 4b and S9a, the perovskite film based on PVSK/CsBr and PVSK/CsBr + Urea exhibits stronger PL intensity compared to that of pristine perovskite film, indicating decreased trap state density and non-radiative recombination (NRR) [37]. Compared with PVSK/CsBr film, the blue-shifted emission peaks of PVSK/CsBr + Urea mainly result from the reduced deep traps in bulk [38, 39]. In addition, the results from time-resolved photoluminescence (TRPL) measurements (Figs. 4c and S9b) showed that the perovskite film based on CsBr + Urea has a longer average carrier lifetime (775.65 ns) than those of PVSK/CsBr (122.4 ns) and pristine perovskite films (19.5 ns), which further demonstrates the suppressed trap-assisted NRR of the perovskite films through Urea synergy with CsBr [40–42].

The effect of Urea in the perovskite was studied to gain deeper insight into the mechanism for the reduced NRR losses. XPS measurement was taken on the perovskite surface for the PVSK/CsBr and PVSK/CsBr + Urea samples. As shown in Fig. 4d, e, the N 1*s* peak at 400.7 eV and the O 1*s* peak at 531.8 eV were considered the characteristic peaks of FA^+^ and OH^−^ on the perovskite surface [43, 44]. The additional peaks at 400.3 and 532.6 eV in N 1*s* and O 1*s* spectra were evaluated as the -NH_2_ and C = O of Urea, respectively, suggesting that Urea was still in the bulk of perovskite. As shown in Fig. 4f, g, the Pb 4*f* and I 3*d* peaks of PVSK/CsBr film shifted toward the low binding energy after being pacified with Urea (0.12 eV for Pb 4*f* and 0.23 eV for I 3*d*, respectively), which was attributed to the interaction between -NH_2_ and C=O of Urea and uncoordinated Pb^2+^ [45, 46].

The energy band structure of PVSK/CsBr and PVSK/CsBr + Urea was determined via ultraviolet photoelectron spectroscopy (UPS) and *E*_g_ values. As shown in Fig. S10, the Fermi level (*E*_F_) of PVSK/CsBr film was calculated from the equation of *E*_F_ = hν—*E*_cutoff_ (hν = 21.2 eV), which shifted from 4.48 to 4.79 eV after adding Urea, indicating better energy alignment between perovskite and electron-transporting layer (ETL) [47]. Kelvin probe force microscopy (KPFM) was performed to investigate their surface potentials, as shown in Fig. 4h, i. The surface of PVSK/CsBr + Urea exhibited a lower average surface potential (223 mV) than that of PVSK/CsBr film (709 mV), confirming the result from the UPS measurement above. The better energy alignment could help avoid carrier accumulation and facilitate efficient charge transfer at the interface between perovskite and ETL, resulting in increased open-circuit voltage (*V*_OC_), as discussed below [45, 48].

### Photovoltaic Performance and Stability

**Fig. 5:**
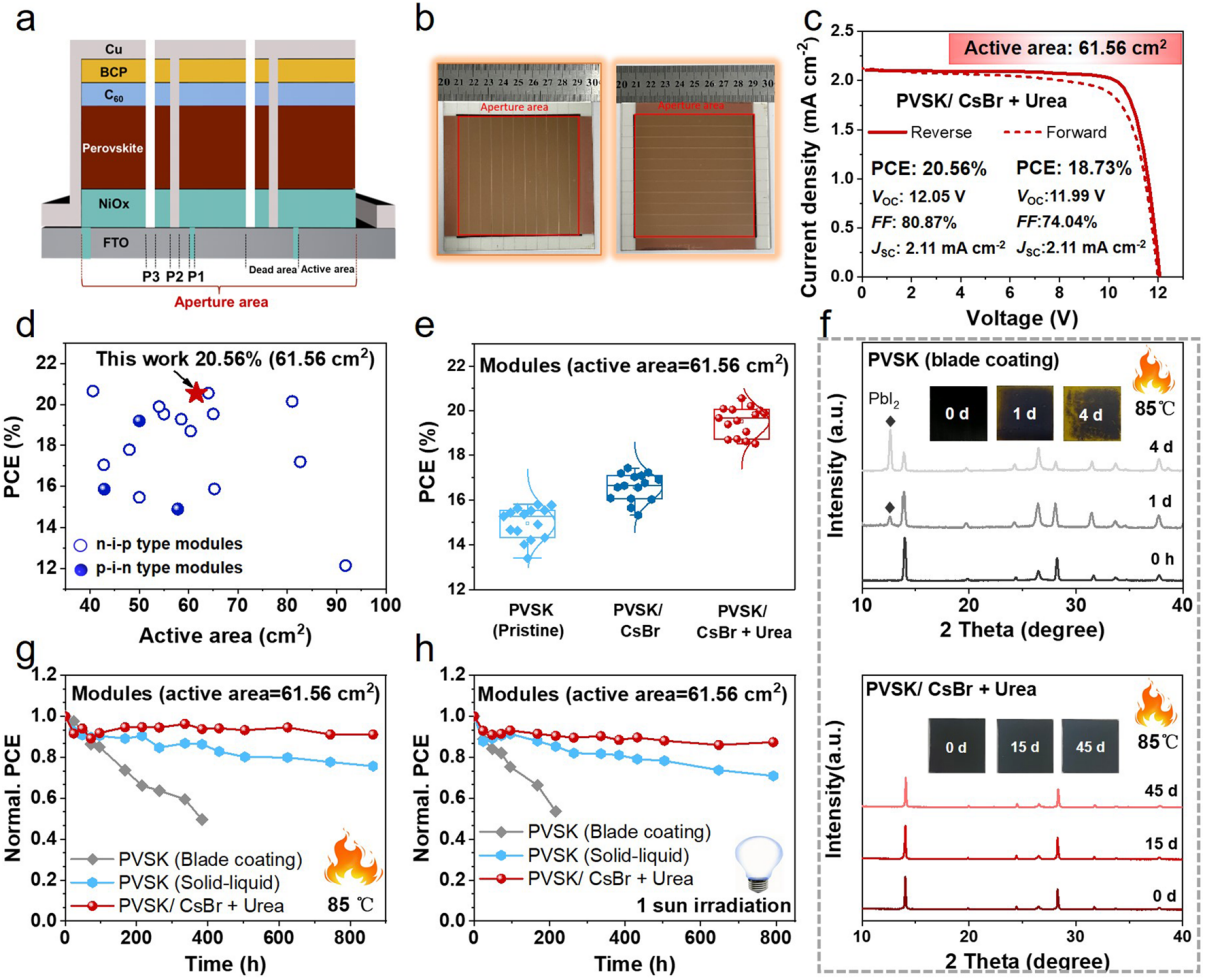
**a** Configuration with a cross-section view of a solar module. **b** Photographs of PSM with a size of 10 × 10 cm^2^. **c** Optimized *J − V* curves of PSCs based on pristine PVSK/CsBr + Urea. **d** Plots of the PCE versus active area for PSMs with *n-i-p* and *p-i-n* type reported in the literature (see Table S3). **e** PCE distribution of 15 modules based on pristine perovskite, PVSK/CsBr, and PVSK/CsBr + Urea. **f** XRD patterns of the blade-coating PVSK and PVSK/CsBr + Urea films stored in the glove box at 85 °C. **g** PCE evolution of the unencapsulated PSMs based on blade-coating PVSK, solid-liquid PVSK, and PVSK/CsBr + Urea stored in the glove box at 85 °C. **h** PCE evolution of the unencapsulated PSCs based on blade-coating PVSK, solid-liquid PVSK, and PVSK/CsBr + Urea stored in N_2_ atmosphere under continuous irradiation (1 sun illumination, white light-emitting diode (LED), 100 mW cm^−2^)

The manufacturing procedure of perovskite film deposited by the solid–liquid two-step method is shown in Fig. S11, and the target perovskite film prepared by the solid–liquid two-step method exhibited more uniformity compared with blade-coating perovskite film (Fig. S12). PSMs with the device structure of FTO/NiO_x_/perovskite/C_60_/BCP/Cu were fabricated (Fig. 5a), and the photographs and structure of the PSMs we prepared are also shown in Figs. 5b and S13. The width of each sub-cell in the PSMs is 0.733 cm, and the dead width for connecting sub-cells is 0.033 cm. Therefore, the GFF (geometric fill factor), the ratio of the active area (61.56 cm^2^) to the aperture area (64.48 cm^2^), is 95.47%. Their *J-V* curves are plotted in Fig. S14, and the parameters are summarized in Table 1. The pristine PSMs based on the solid–liquid two-step method show a PCE of 15.81%, which was higher than that of the blade-coated device (13.47%) due to the better crystallinity. The PSM based on PVSK/CsBr exhibited a PCE of 17.43% with enhanced *V*_OC_ (11.25 V) and *FF* (78.39%), which is attributed to the modification of NiO_x_ and higher-quality perovskite film. As shown in Fig. 5c, after the coordinated regulation of the perovskite buried interface and crystal growth by CsBr and Urea, the corresponding device performance was significantly improved. The PSMs based on PVSK/CsBr + Urea achieved a PCE of 20.56% with a *J*_SC_ of 2.11 mA cm^−2^, *V*_OC_ of 12.05 V, and *FF* of 80.87% with an active area of 61.56 cm^2^, which is the best performance for the *p-i-n* type PSMs with active area > 40 cm^2^ reported in the literature (Fig. 5d and Table S3). The preferable crystal growth orientation and enlarged grain size were conducive to charge transporting, which were responsible for the increased *J*_SC_. The significantly enhanced *V*_OC_ and *FF* of the device based on CsBr + Urea were mainly attributed to the superior energy level alignment and suppressed defects in the perovskite layer. The integrated *J*_SC_ derived from EQE spectra were 1.80, 1.94, and 2.07 mA cm^−2^ for the vapor-deposited PVSK, PVSK/CsBr, and PVSK/CsBr + Urea device, respectively (Fig. S15), which were close to their measured *J*_SC_. In addition, the PSMs based on PVSK/CsBr and PVSK/CsBr + Urea exhibited good repeatability with a PCE_average_ of 19.53% and 16.57%, respectively (Figs. 5e and S16), much better than that of pristine PVSK (PCE_average_ = 14.97%).

**Table 1:** Photovoltaic parameters of PSMs based on PVSK (blade coating), PVSK (solid–liquid), PVSK/CsBr, and PVSK/CsBr + Urea

Sample	*V*_oc_ [V]	*J*_sc_ [mA cm^−2^]	FF [%]	PCE [%]	PCE_average_ [%]
PVSK (blade coating)	11.04	1.81	67.43	13.47	12.77
PVSK (solid–liquid)	11.14	1.86	76.34	15.81	14.97
PVSK/CsBr	11.25	1.98	78.39	17.43	16.57
PVSK/CsBr + Urea	12.05	2.11	80.87	20.56	19.53

The moisture stability of the perovskite films was investigated in ambient air (relative humidity of 35 ± 5%) and tracked by XRD measurements. As shown in Fig. S17, the diffraction peak at 12.9° is assigned to the characteristic peak of PbI_2_, which was observed for blade-coating perovskite film, and the diffraction intensity increases along with the storage time. In contrast, no obvious PbI_2_ signals existed in PVSK/CsBr and PVSK/CsBr + Urea films after 45 d. The thermal stability of the corresponding films was performed under continuous heating at 85 °C (Figs. 5f and S18). There was an obvious PbI_2_ peak for blade-coating perovskite film after 4d, and the perovskite films showed significant decomposition. In comparison, there were no obvious changes in corresponding PVSK/CsBr and PVSK/CsBr + Urea films after 45 days. These results proved the promoted environment and thermal stability of PVSK/CsBr and PVSK/CsBr + Urea films with solid–liquid two-step deposition strategy.

The long-term stability of PSMs was further explored (Figs. 5g, h and S19). As illustrated in Fig. S19, the aging tests of the encapsulated PSMs were stored under a relative humidity of 75 ± 5%. The PSMs with solid–liquid two-step deposition strategy (pristine PVSK and PVSK/CsBr + Urea) exhibited excellent stability, maintaining 73% and 89% of their original PCE after 800 h for pristine PVSK and PVSK/CsBr + Urea, respectively. By comparison, the PCE of the blade-coating PSM reduces to below 50% of its original value after 432 h. The thermal aging test showed the unencapsulated PSMs based on pristine PVSK and PVSK/CsBr + Urea could sustain over 75% and 91% of its original PCE after being stored at 85 °C in N_2_ for 864 h, while the PCE of the blade-coating PSM retained below 50% of its initial value after 384 h (Fig. 5g). Moreover, the long-term stability under continuous light soaking (white LED, 100 mW cm^−2^) in a glove box was further investigated. The PSMs with a solid–liquid two-step deposition strategy can maintain 71% and 87% of their original PCE after 792 h for pristine PVSK and PVSK/CsBr + Urea, respectively. However, the PSM based on blade-coating PVSK dropped to 54% of its premier value after storing 216 h (Fig. 5h). The enhanced performance and stability of the PSMs based on the PVSK/CsBr + Urea should be responsible for the improved intrinsic stability of the perovskite film and restrained defect density at the buried interface and perovskite grain boundary. In short, the optimized deposition process, modified buried interface, and additive strategy accounted for the preferable crystal growth orientation and enlarged perovskite grain size with increased carrier lifetime and reduced NRR, resulting in the high photovoltaic performance and superior stability of target PSMs.

## Conclusions

This work demonstrated a solid–liquid two-step film formation technique to prepare uniform large-area perovskite films. The targeted modification for the NiO_x_/perovskite buried interface and the introduction of Urea additives were utilized to reduce interface recombination and regulate perovskite crystallization, achieving a large-area perovskite film possessing larger grains, fewer pinholes, and reduced defects. Finally, the inverted PSM with an active area of 61.56 cm^2^ achieved a champion PCE of 20.56%. More encouragingly, it also promoted the stability of corresponding devices under light, heat, and moisture conditions. We firmly believe that our strategy via solid–liquid two-step film formation will open a new avenue for highly efficient and stable large-area PSMs and promote the commercialization of this technology.

## Supplementary Information

Below is the link to the electronic supplementary material.

Supplementary file1 (PDF 1705 KB)
