# Supplementary material for: Efficient and Stable Inverted Perovskite Solar Modules Enabled by Solid–Liquid Two-Step Film Formation
Source: Nanomicro Lett. 2024 May 2;16:190. doi: 10.1007/s40820-024-01408-2 (PMC11065817; doi:10.1007/s40820-024-01408-2)
Supplement: Supplementary file 1 — Supplementary file1 (PDF 1705 KB) [file 40820_2024_1408_MOESM1_ESM.pdf]

Supporting Information

Solid-liquid Two-step Film Formation Enables Efficient and Stable Inverted Perovskite Solar Modules

1. Materials

All the chemicals were purchased from commercial businesses without further purification. Formamidinium iodide (FAI) (98%, Greatcell Solar Materials Pty Ltd), Lead iodide (PbI2) (99.99%, Zhejiang Yitai Technology Co., Ltd.), Urea (99.999%, Aladdin), Bathocuproine (BCP) (>99.0%, TCI), Fullerene (C_{60}) (99.9%, Tanfeng Tech. Inc.). Copper particle (Cu) (99.999%, Fuzhou Yingfeixun Photoelectric Technology Co., Ltd). Isopropanol (IPA) was purchased from Sigma-Aldrich. Cesium bromide (CsBr) (99.9%) and methylammonium chloride (MACl) (99.5%) were purchased from Xi'an Polymer Light Technology in China. All the materials are stored in the nitrogen-filled glove box to avoid the water.

2. Measurements

X-ray diffraction (XRD) measurements were carried out using a Rigaku Ultima IV X-ray powder diffractometer with Cu Kα radiation over the 2θ = 10−60°. UV-Vis absorption spectra were measured on a UV-2600 spectrometry (Shimadzu). SEM images were taken on Zeiss Gemini 300. For SEM characterization, the accelerating voltage of the electron beam is 3 kV. AFM images were obtained using Bruker Multimode 8 Atomic Force Microscopy. Thermo ESCALAB 250XI was used for UPS and XPS measurements. Steady-state photoluminescence (PL) and time-resolved photoluminescence (TRPL) were measured via a Spectrofluorometer FS5 (Edinburgh Instruments Ltd.) with excitation at 510 nm. Fitted TRPL decay provides

insights into the charge carrier dynamics, which was fitted using an empirical biexponential equation:[48]

$$y = A_1 \exp\left(\frac{-t}{\tau_1}\right) + A_2 \exp\left(\frac{-t}{\tau_2}\right) + y_0$$

The average PL lifetime is determined by the following equation:

$$\tau_{avg} = \frac{A_1 \tau_1{}^2 + A_2 \tau_2{}^2}{A_1 \tau_1 + A_2 \tau_2}$$

where y_0 is the decay constant, A_1 and A_2 are decay amplitudes, τ_1 is the fast decay process associated with the charge transfer, and τ_2 is the slow decay process resulting from charge recombination.

3.1 In-situ photoluminescence measurements

In-situ photoluminescence (in-situ PL) spectra were characterized by home-built equipment, including an excitation system, fiber system, and detector system. The testing samples are held in a temperature-controlled N2 glove box or humidity-controlled air box with a fiber system set around the sample, while the excitation and detection system is set in the ambient environment connected with the fiber. An excitation system was used using an excitation laser (315 nm, max = 300 W). Excitation light was introduced to the sample through a fiber. The emitted light from the sample was collected by fiber and introduced to a spectrophotometer (Ocean Optics USB2000). A 550 nm low pass filter is applied in the light pass to the spectrophotometer.

3.2 Perovskite photovoltaic mini-modules fabrication and characterization.

For the large-size perovskite modules, laser etching, including P1, P2, and P3 processes, was conducted by a nanosecond laser (ZNLB-22V1-LW300). Before use, the FTO was cleaned with ultraviolet ozone for 15min. The following procedures were fabricated on the pre-patterned large FTO glass substrates. The mini-modules were fabricated on the pre-patterned

large FTO glass substrates (10×10 cm). For the P1 process,10 cm × 10 cm size FTO substrates were patterned with a scribing width of 35 μm with 11-strip connected in series. The NiO_x films were prepared by magnetron sputtering at 9×10^{-4} Pa, and the power was controlled at 500 W for 300s, and the thickness was about 25 nm. The first step for the PSM fabrication is different for the blade-coating and solid-liquid process. For the blade-coating process, the solution of PbI_2 (461 mg) in 1mL DMF: DMSO (10:1) was blade-coated onto the above substrate at a movement speed of 20 mm s^{-1} in air. For the solid-liquid process, CsBr (15 nm) and PbI_2 (300nm) were deposited sequentially by thermal evaporation on the NiO_x substrates. For the second step, the solution of FAI: MACl (110 mg: 11 mg) in the absence or the presence of Urea in 1 mL IPA was blade-coated onto the above PbI_2-covered FTO glass substrates at a movement speed of 15 mm s^{-1} in air. The N_2 knife worked at 0.5 kaf cm^{-2} during blade-coating. Then, the film was annealed at 150 °C for 20 min in air with a relative humidity of 40 ± 5%. Afterward, 25 nm C_{60}, 5 nm BCP, and 240 nm copper were sequentially deposited sequentially using thermal evaporation under a high vacuum (≤8×10^{-4} Pa). For the P3 process, the Cu layer was scribed with a 95 μm width. A full structure of the large-size PSC is shown in Fig. S13. The fabricated modules typically have 11 sub-cells, each with a width of 7.33 mm. The total dead width was 0.332 mm, giving a GFF of 95.47%.

The mini-modules were fabricated on the pre-patterned large FTO glass substrates (10×10 cm, P1 width 35 μm) following the same procedure as the solar cells. The fabricated modules typically have 11 sub-cells, each with a width of 7.33 mm. The laser scribing was performed twice with a laser marker. The final widths of P2 and P3 were measured to be 116 and 95 μm, respectively. The total scribing line width was 0.332 mm, giving a GFF of 95.47%.

The current density–voltage (*J–V*) characteristics of the mini-modules were measured using a Keithley 2400 Source Meter under standard AM1.5 G illumination, and the light intensity was calibrated using a standard silicon reference cell (Newport, Oriel Sol3Atm). The *J-V* curves were measured by forward scan (-0.1 to 13 V) and reverse scan (13 to -0.1 V). EQE spectra were obtained with a PVE300-IVT QE measurement kit by focusing a monochromatic light beam onto the devices.

4. Supplementary Figures.

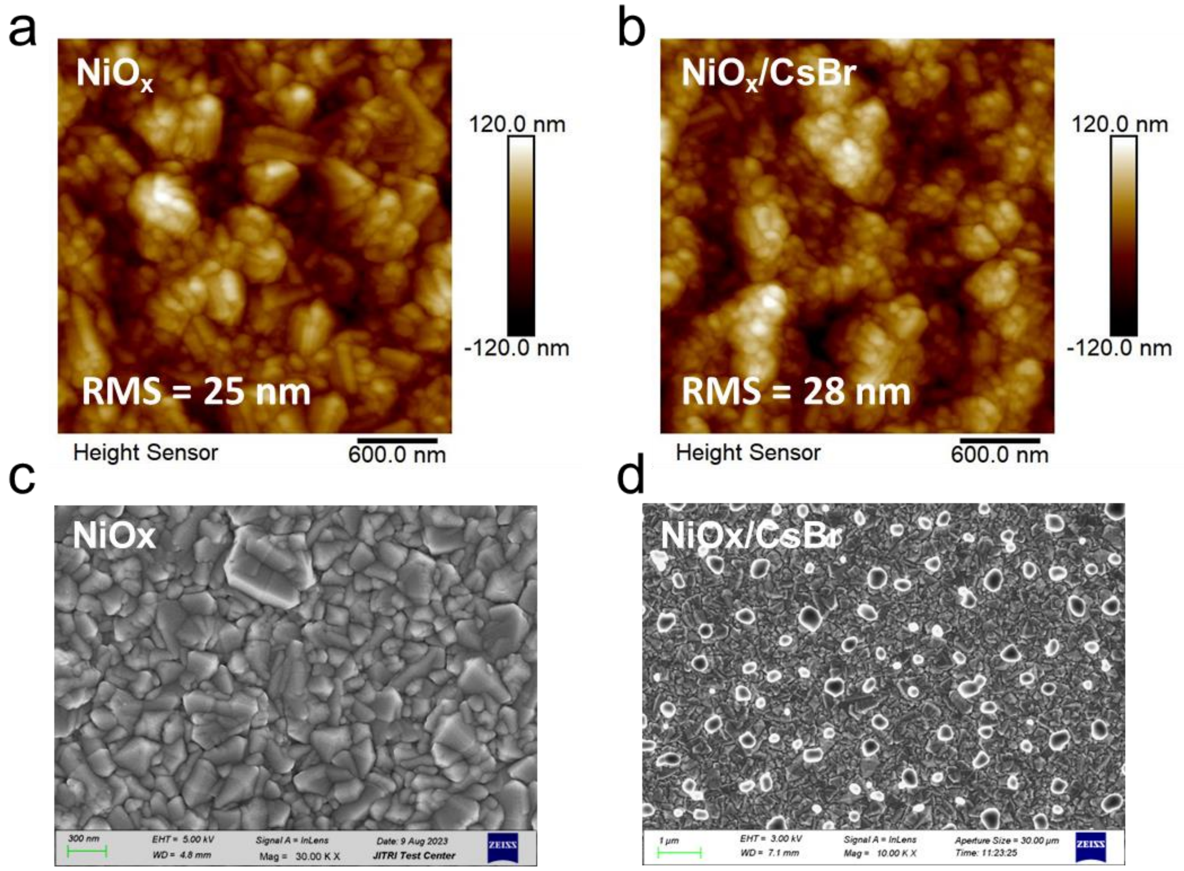

Fig. S1. AFM characterization(a,b) and top-view SEM results(c,d) of NiO and NiO/CsBr.

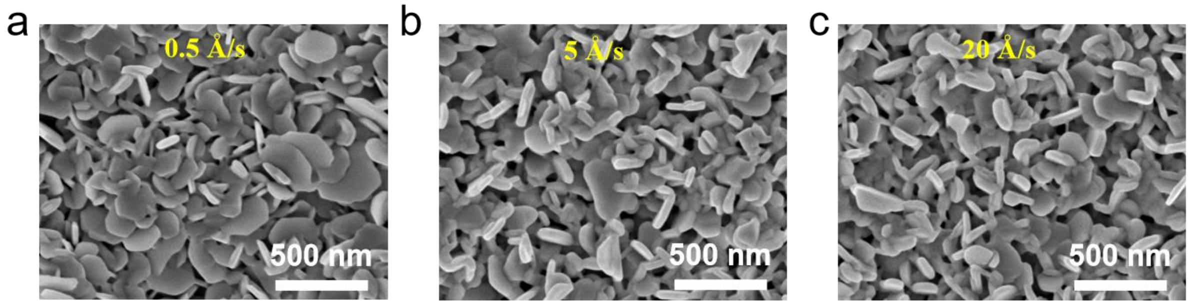

Fig. S2. The SEM images of PbI_2 prepared by vapor deposition with different rates.

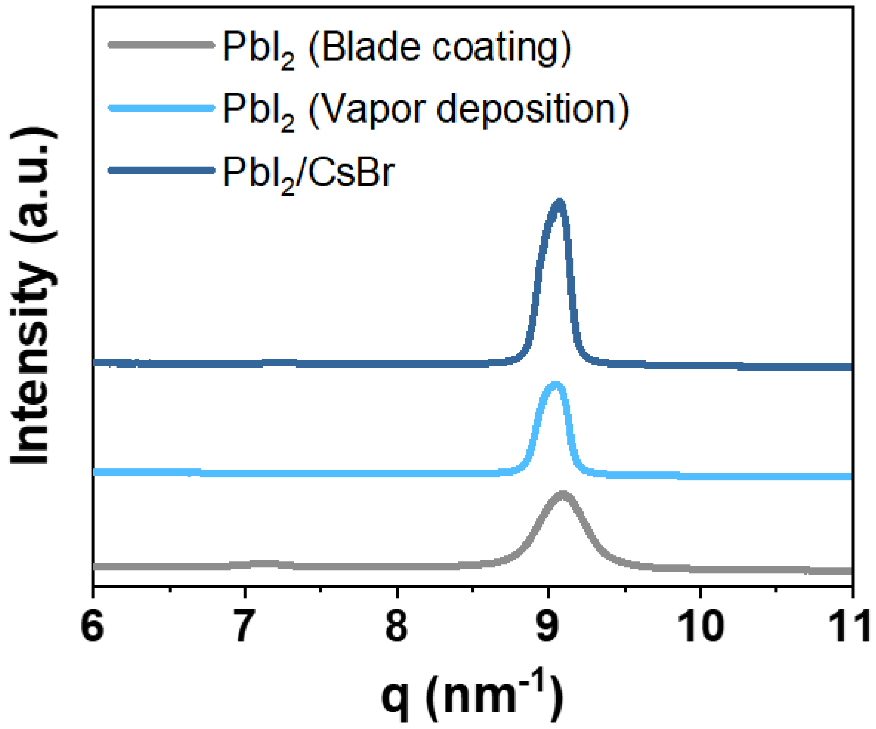

Fig. S3. The GIWAXS line-cut profiles of PbI_2 films (deposited by blade coating and vapor) and $PbI_2/CsBr$ film.

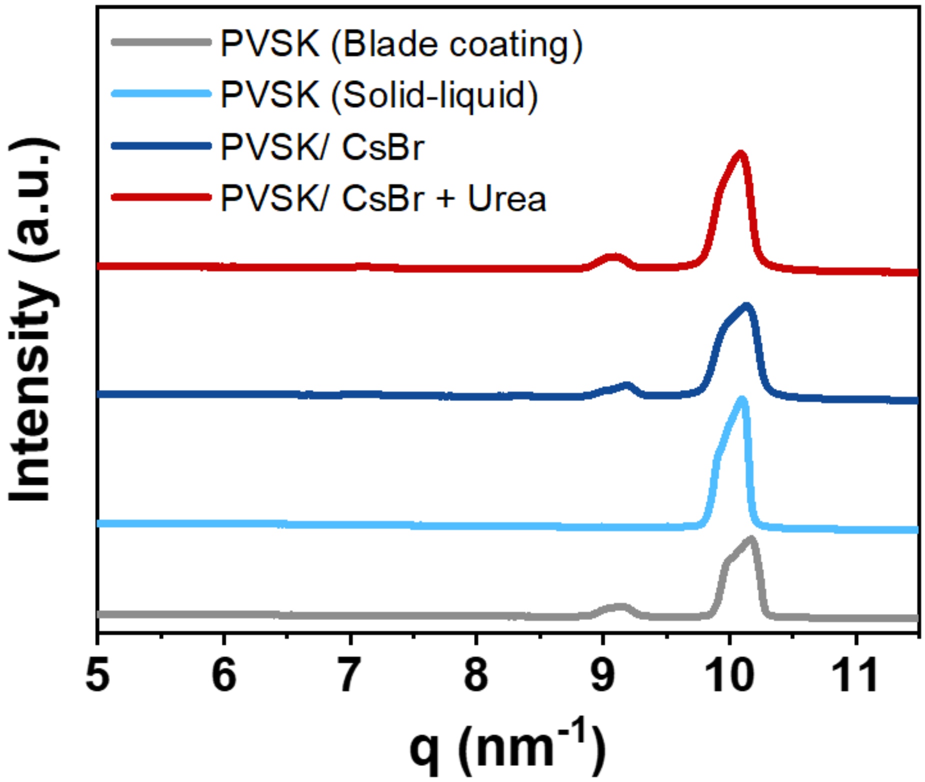

Fig. S4. The GIWAXS line-cut profiles of perovskite films (prepared by blade coating and solid-liquid deposition), PVSK/CsBr and PVSK/CsBr + Urea films.

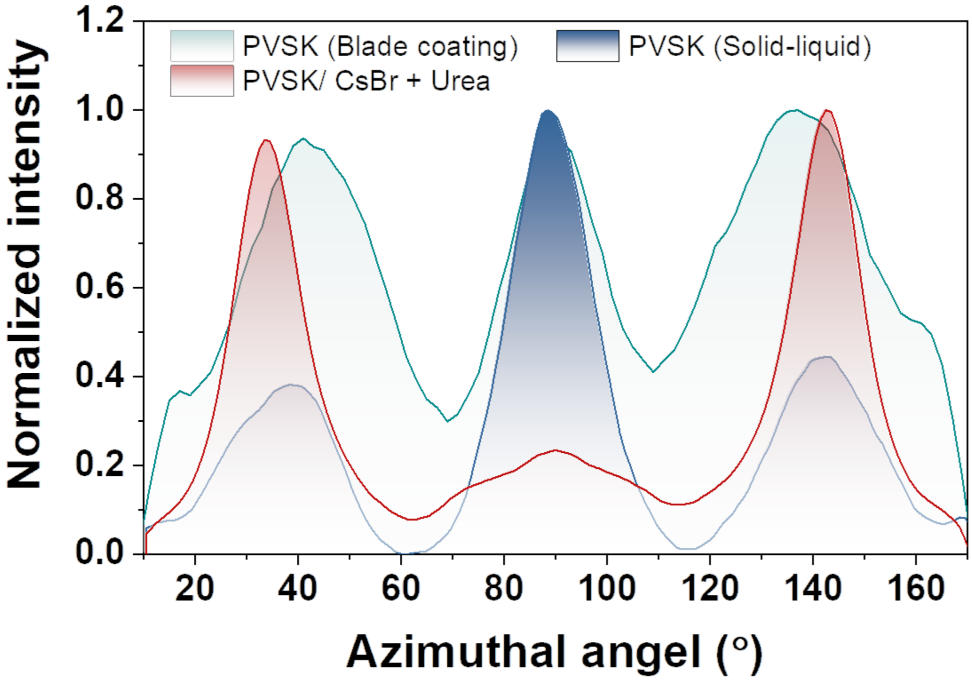

Fig. S5. Normalized integrated intensities of the perovskite (100) planes as marked in Fig. 3a-3c and plotted as a function of the azimuthal angle.

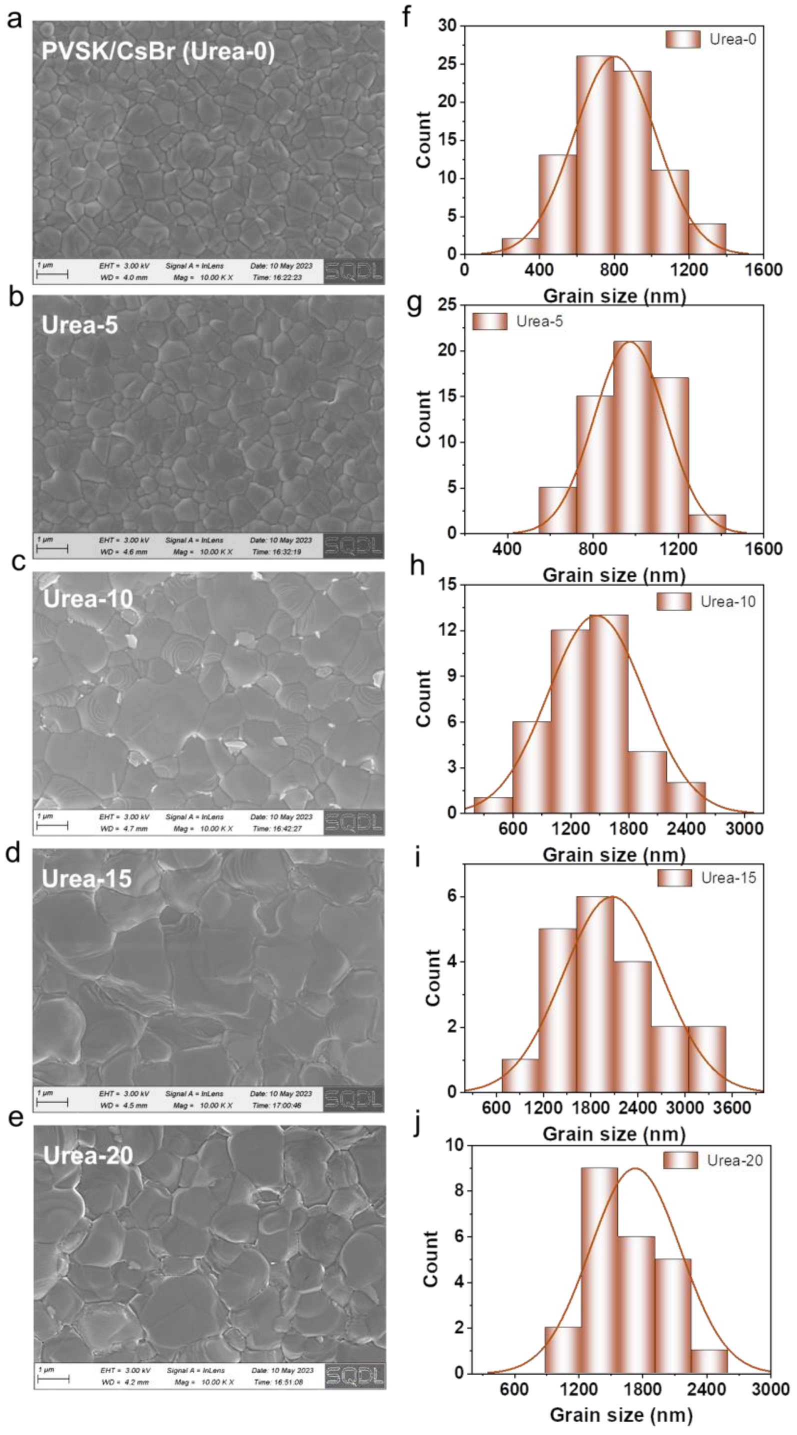

Fig. S6. The SEM images and grain size distribution of PVSK/CsBr + Urea film with varying concentrations.

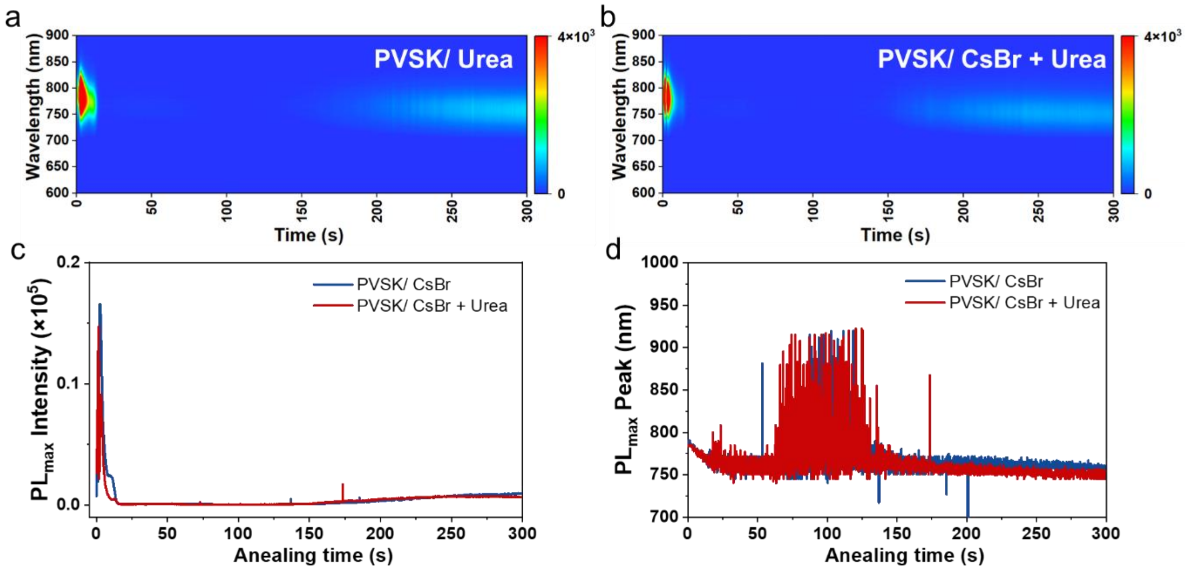

Fig. S7. Heat maps of in situ PL for (a) PVSK/CsBr and (b) PVSK/CsBr + Urea within 300s. (c) Evolution of the intensity of maximum peak extracted from in-situ PL spectra during the annealing process. (d) The shift of the wavelength corresponding to the maximum peak of the in-situ PL spectra.

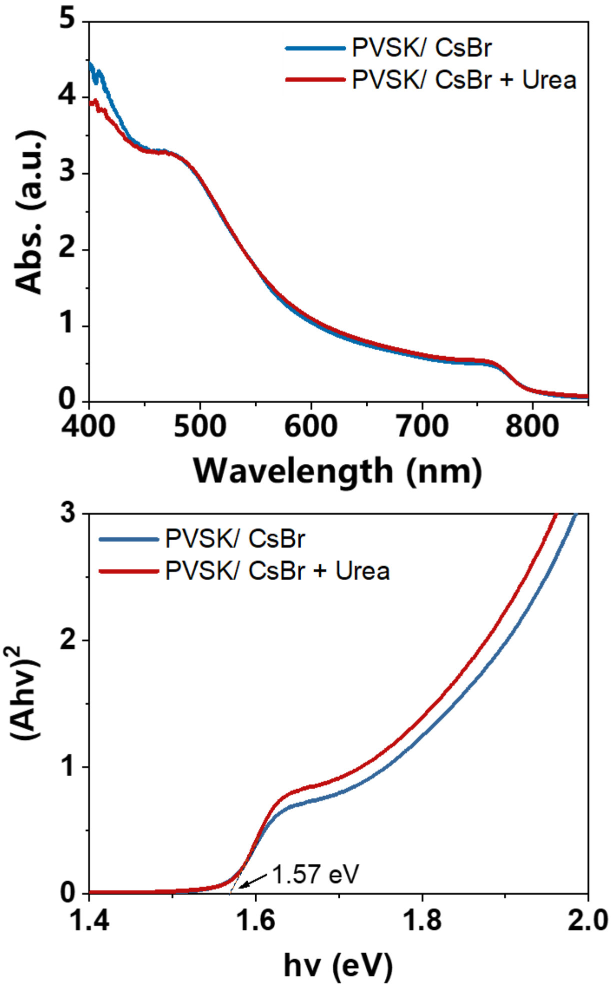

Fig. S8. (a) The UV-vis absorption spectrum of perovskite films and (b) the corresponding Tauc plots based on PVSK/CsBr and PVSK/CsBr + Urea.

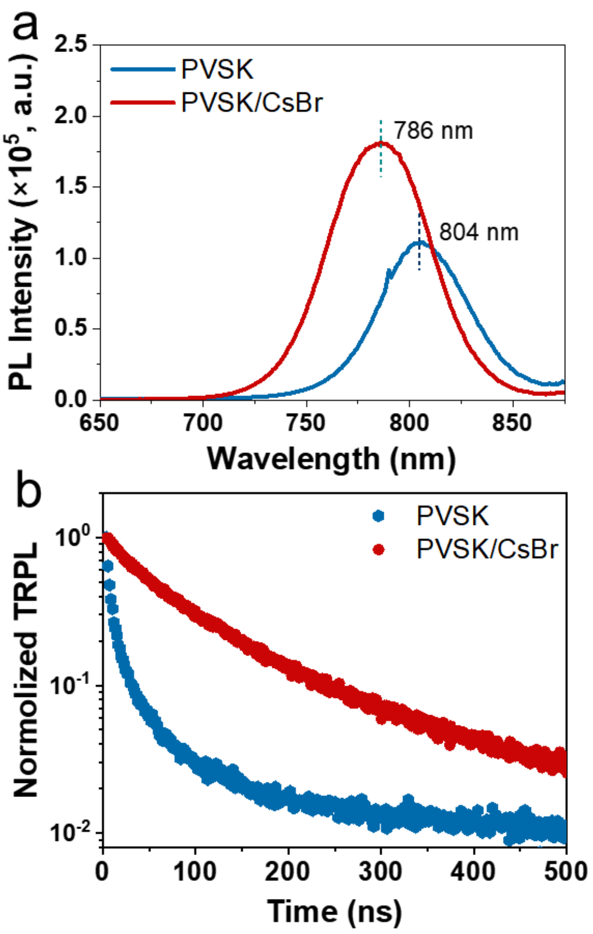

Fig. S9. The photoluminescence (PL) and (c) time-resolved photoluminescence (TRPL) spectra of the pristine PVSK and PVSK/CsBr films.

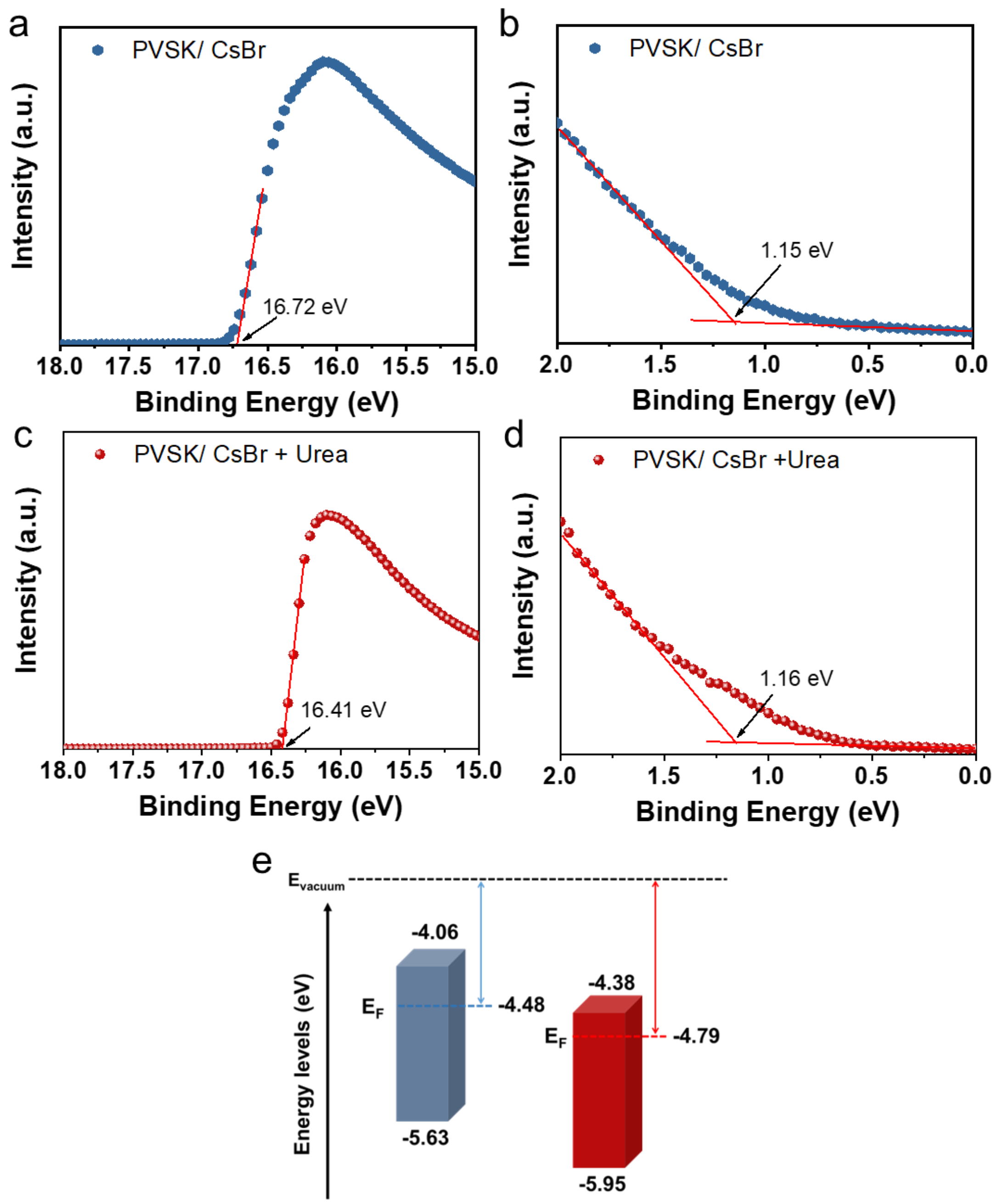

Fig. S10. UPS spectra of (a, b) PVSK/CsBr and (c, d) PVSK/CsBr + Urea. (e) The schematic diagram of the energy level arrangement of PVSK/CsBr and PVSK/CsBr + Urea.

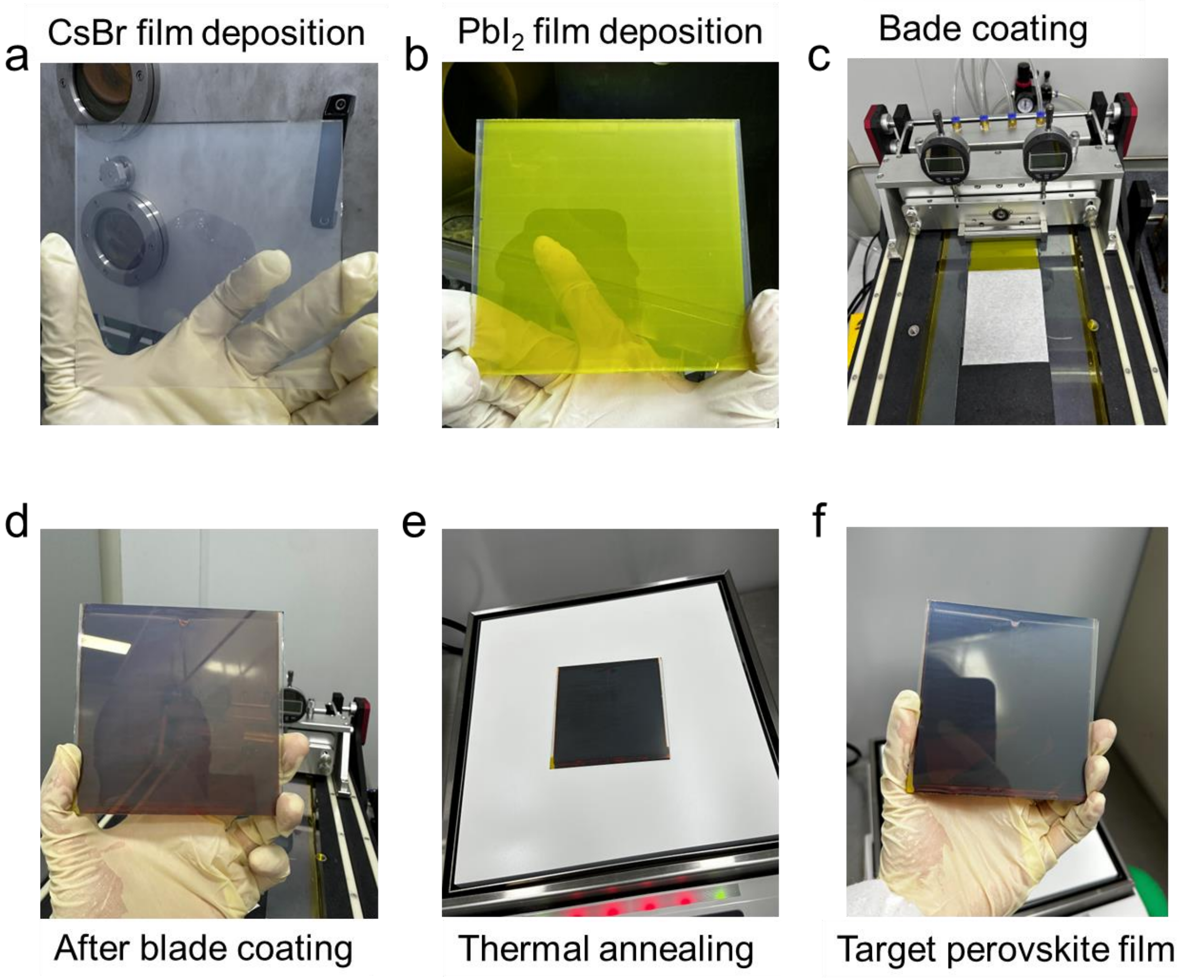

Fig. S11. The manufacturing procedure of perovskite film deposited by the solid-liquid two-step method.

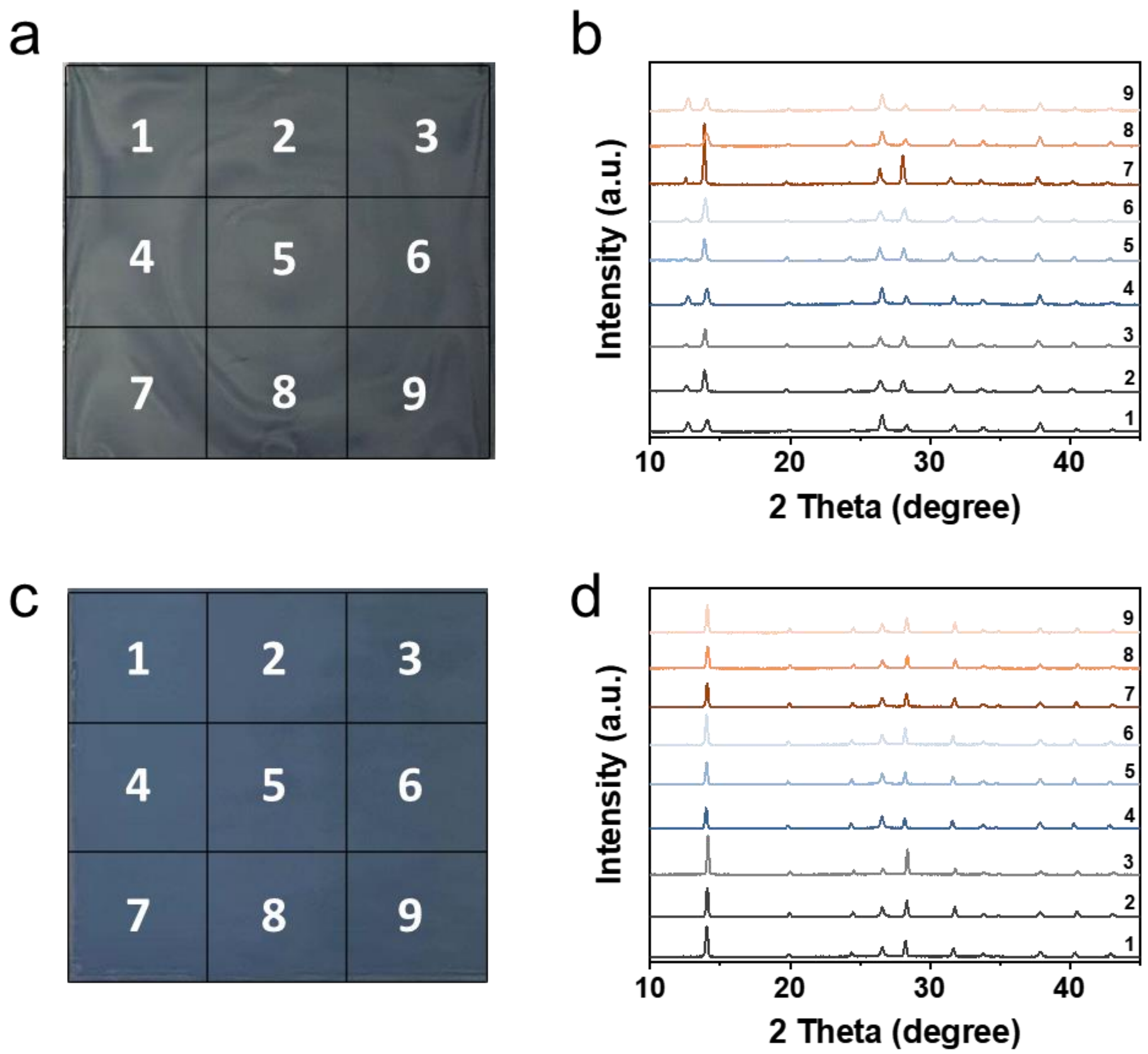

Fig. S12. The photos and XRD patterns of nine-portion perovskite films prepared by (a, b) blade coating and (c, d) solid-liquid deposition.

lines.

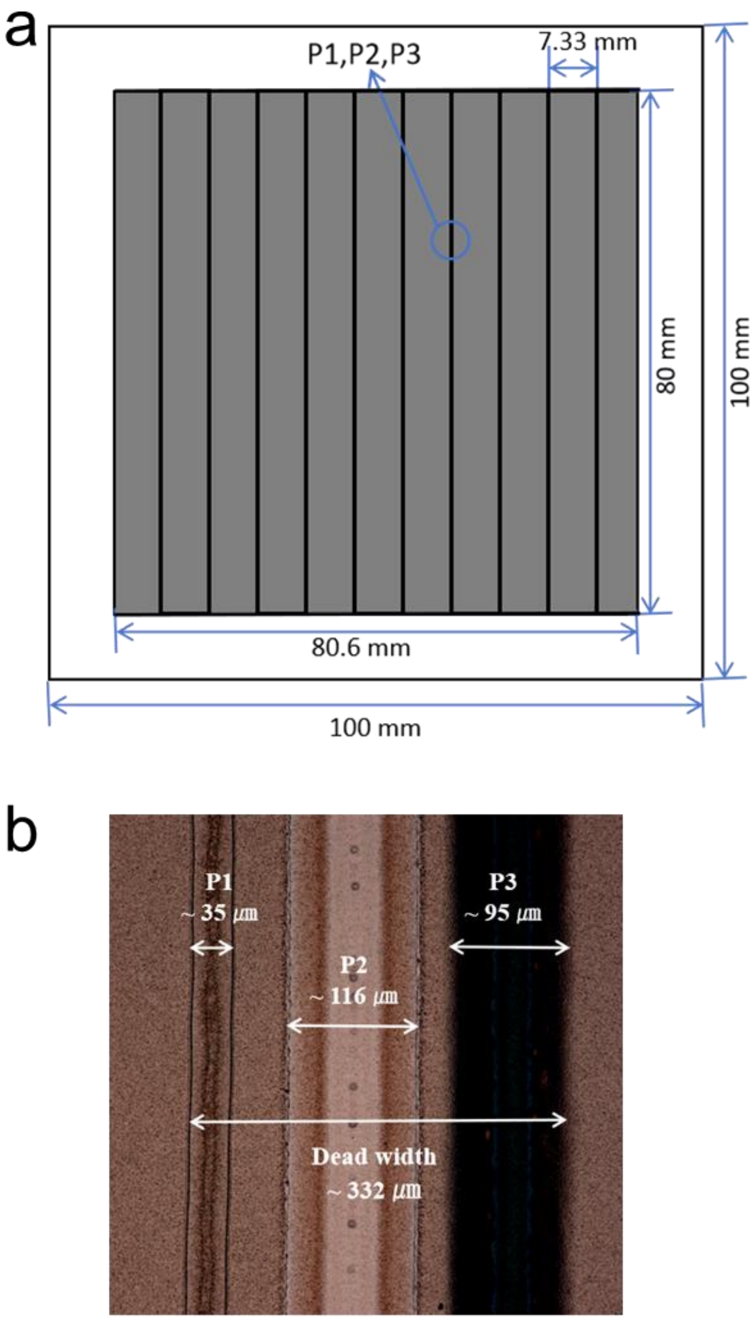

- Active width=Subcell width-Deed width= 7327-332=6995
- GFF=Active width/Subcell width=6995/7327=95.47%
- Aperture area =8.06×8=64.48cm^2
- Active area=Aperture area×GFF=61.56 cm^2

Fig. S13. (a) size of a 10 cm × 10 cm^2 based PSM. (b) Optical micrograph of P1, P2 and P3 lines.

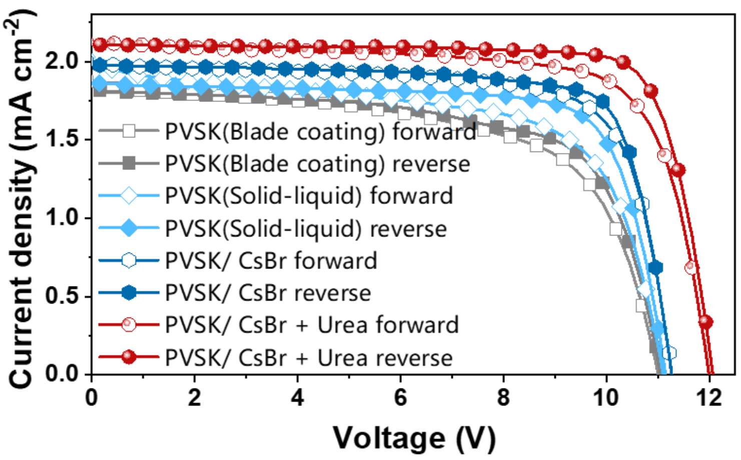

Fig. S14. (a) The *J-V* curves of best-performing PSMs based on PVSK (Blade-coating), PVSK (Solid-liquid), PVSK/CsBr and PVSK/ CsBr + Urea.

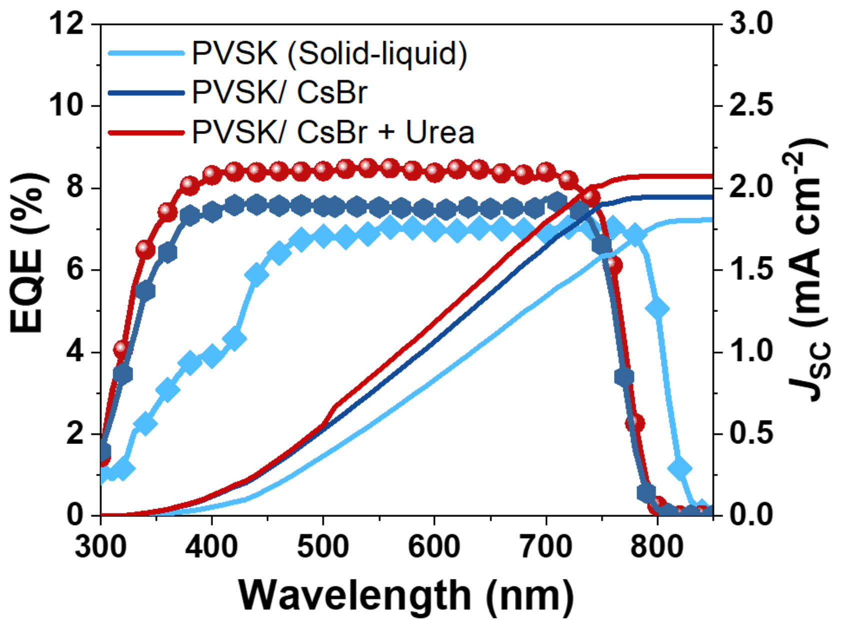

Fig. S15. The EQE curves of best-performing PSMs based on pristine PVSK, PVSK/CsBr and PVSK/CsBr + Urea.

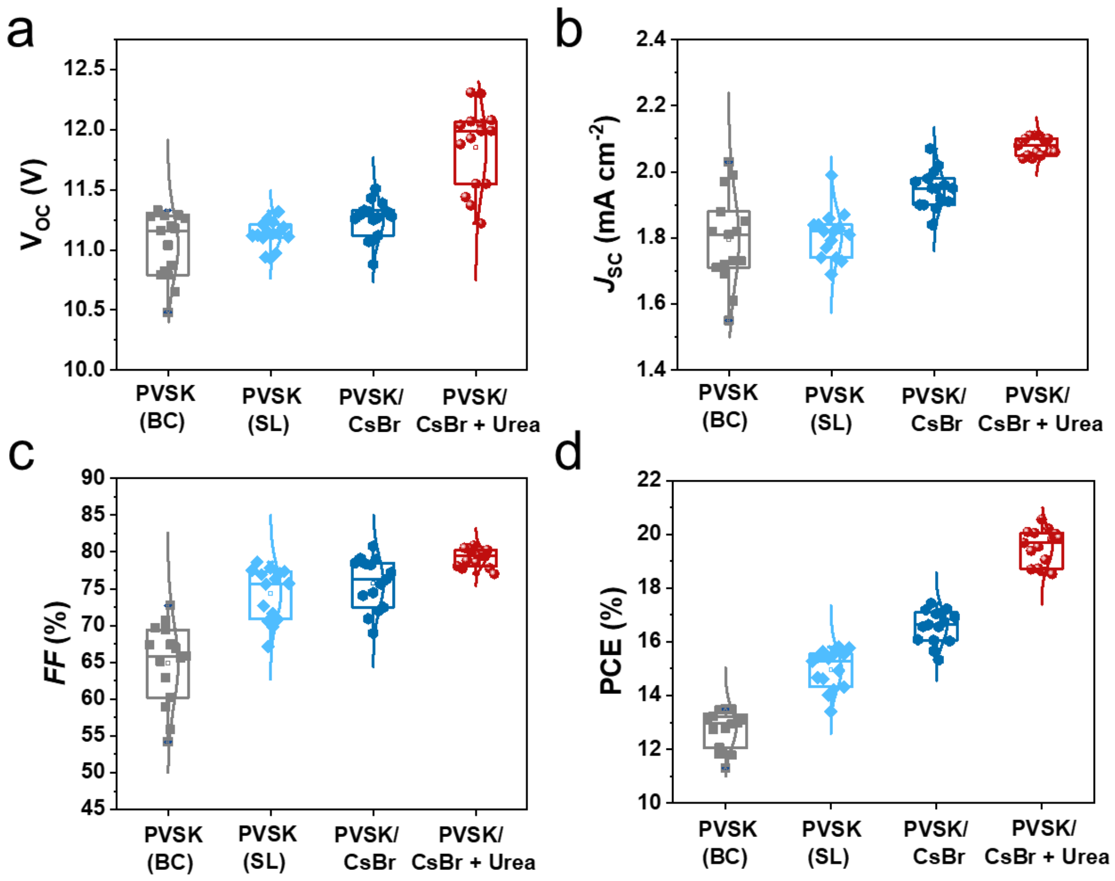

Fig. S16. (a-d) Photovoltaic performance variations of (a) V_{OC}, (b) J_{SC}, (c) FF and (d) PCE for PSMs based on PVSK (Blade coating), PVSK (Solid-liquid), PVSK/CsBr and PVSK/ CsBr + Urea.

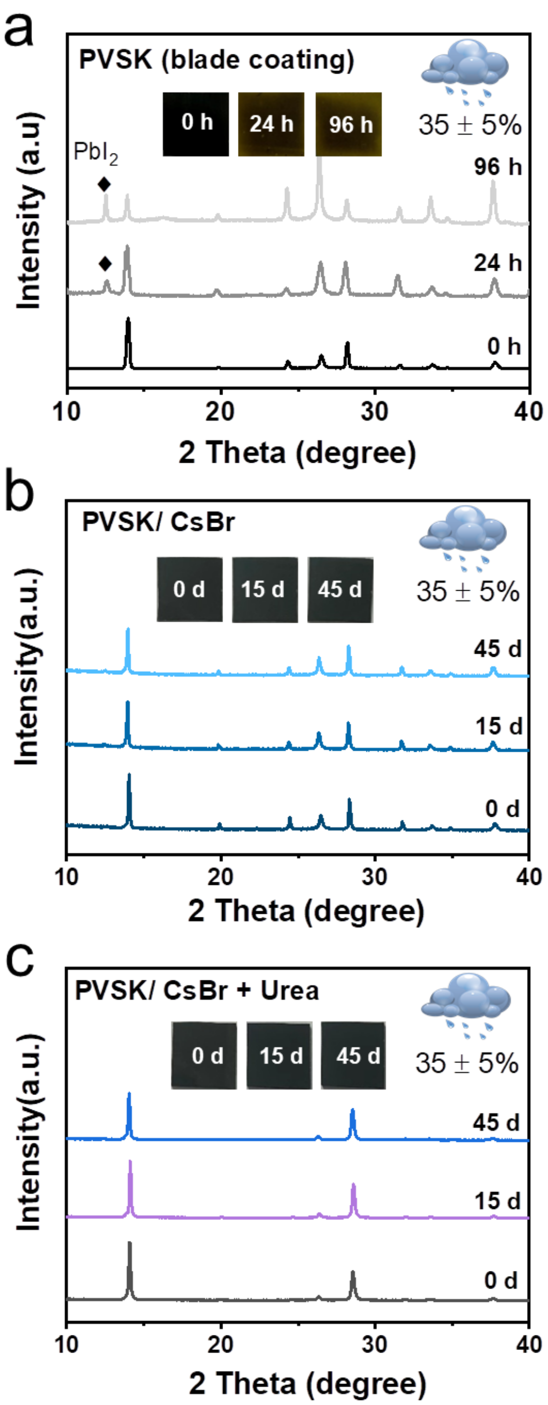

Fig. S17. The XRD patterns of (a) PVSK (Blade coating), (b) PVSK/CsBr and (c) PVSK/ CsBr + Urea films stored in ambient air with 35±5% RH under natural light.

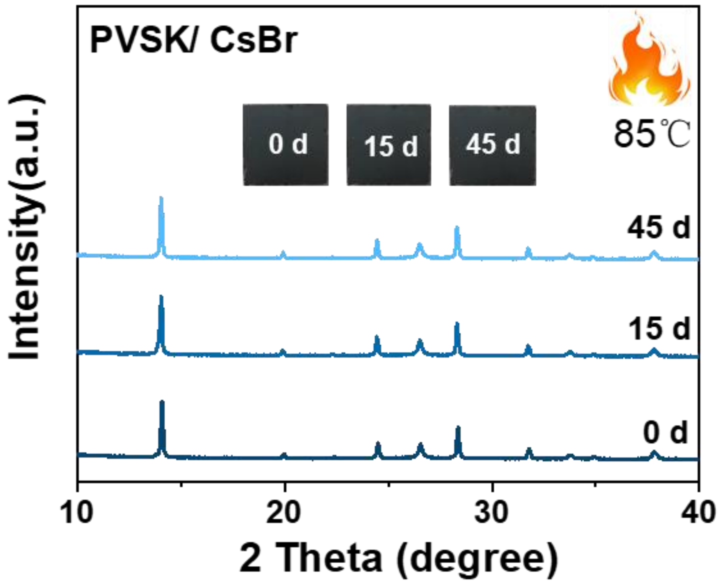

Fig. S18. The XRD patterns of PVSK/CsBr films stored in the glove box at 85 °C.

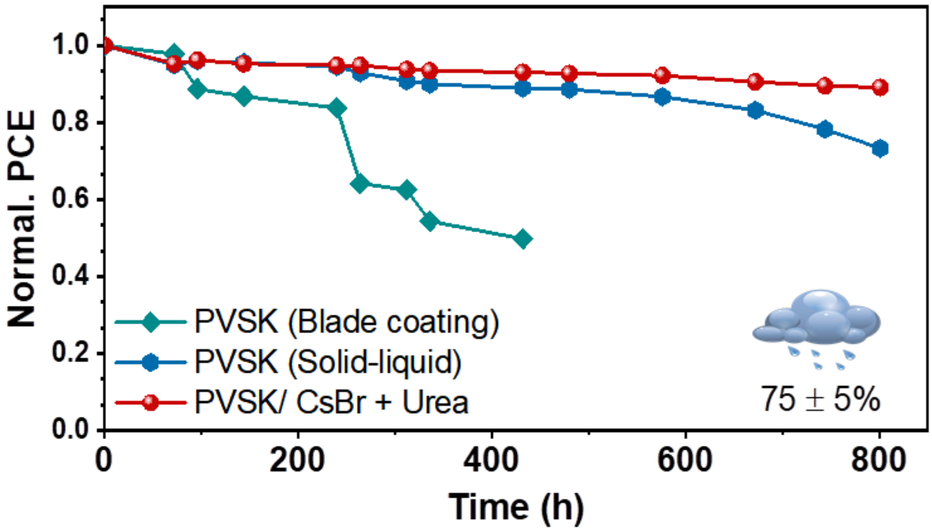

Fig. S19. PCE evolution of the encapsulated PSMs based on blade-coating PVSK, solid-liquid PVSK and PVSK/CsBr + Urea stored in air at room temperature with relative humidity (RH) of 75± 5%.

5. Supporting Tables.

Table S1. Photovoltaic parameters of PVSK based on solid-liquid with different deposition rates.

Deposition rate (0.5 Å s^{-1})	V_{oc} [V]	J_{sc} [mA cm^{-2}]	FF [%]	PCE [%]
0.5	11.10	1.83	76.92	15.64
5	11.14	1.86	76.34	**15.81**
20	11.02	1.85	77.27	15.78

Table S2. Photovoltaic parameters of PVSK/ CsBr + Urea with different content.

Content (mg mL^{-1})	V_{oc} [V]	J_{sc} [mA cm^{-2}]	FF [%]	PCE [%]
Urea-0	11.25	1.98	78.39	17.43
Urea-5	11.51	2.00	78.79	18.18
Urea-10	11.87	2.03	78.90	19.03
Urea-15	12.05	2.11	80.87	20.56
Urea-20	11.52	2.01	77.51	17.92

Table S3. Photovoltaic performance parameters of perovskite solar modules include *p-i-n and n-i-p- type* devices.

Device structuture	Active area [cm^2]	V_{oc} [V]	J_{sc} [mA cm^{-2}]	FF [%]	PCE [%]	Reference
n-i-p	91.8	13.74	1.39	53.87	12.14	Adv. Energy Mater. 2021, 11, 2003712
n-i-p	50	10.46	2.45	60	15.46	Nano-Micro Lett. (2023) 15:182
n-i-p	65.22	13.92	1.514	75.3	15.87	Energy Environ. Sci., 2021, 14, 4903–4914
n-i-p	42.8	16.05	1.498	70.89	17.05	Nano Energy 82(2021) 105685
n-i-p	82.62	10.06	2.54	67.29	17.2	Nano Energy 95 (2022) 107019
n-i-p	48	16.62	22.07	74.65	17.79	Adv. Energy Mater. 2022, 12, 2103420
n-i-p	60.4	11.13	2.35	71.51	18.7	Nano-Micro Lett. (2023) 15:114
n-i-p	58.5	13.7	1.86	75.04	19.28	Adv. Mater. 2023, 2210176
n-i-p	55	15.26	1.79	71.49	19.53	Joule 7, 1–18, July 19, 2023
n-i-p	65	15.35	1.67	76.1	19.54	Science 372, 1327–1332 (2021)
n-i-p	54	15.516	1.79	71.65	19.9	Energy Environ. Sci., 2022, 15, 4404–4413
n-i-p	81	15.46	1.71	76.3	20.15	Adv. Energy Mater. 2023, 2300595
n-i-p	64	18.43	1.457	76.6	20.56	Science 375, 302–306 (2022)
n-i-p	40.6	13.27	2.11	73.8	20.66	Adv. Mater. 2023, 2211593
p-i-n	42.9	13.14	19.71	73.5	15.86	Adv. Energy Mater. 2020, 10, 1903108
p-i-n	57.8	17.24	1.25	68.9	14.9	Nature Energy 3(2018),560–566
p-i-n	50	16.07	1.527	78	19.2	Science 373, 902–907 (2021)
p-i-n	**61.56**	**12.05**	**2.11**	**80.87**	**20.56**	**In this work**
